# A Not-So-Grim Tale: How Childhood Family Structure Influences Reproductive and Risk-Taking Outcomes in a Historical U.S. Population

**DOI:** 10.1371/journal.pone.0089539

**Published:** 2014-03-05

**Authors:** Paula Sheppard, Justin R. Garcia, Rebecca Sear

**Affiliations:** 1 London School of Hygiene and Tropical Medicine, Keppel Street, London, United Kingdom; 2 The Kinsey Institute for Research in Sex, Gender, and Reproduction, Indiana University, Bloomington, Indiana, United States of America; 3 Department of Gender Studies, Indiana University, Bloomington, Indiana, United States of America; University of Arkansas, United States of America

## Abstract

Childhood family structure has been shown to play an important role in shaping a child's life course development, especially in industrialised societies. One hypothesis which could explain such findings is that parental investment is likely to be diluted in families without both natural parents. Most empirical studies have examined the influence of only one type of family disruption or composition (e.g. father absence) making it difficult to simultaneously compare the effects of different kinds of family structure on children's future outcomes. Here we use a large, rich data source (n = 16,207) collected by Alfred Kinsey and colleagues in the United States from 1938 to 1963, to examine the effects of particular childhood family compositions and compare between them. The dataset further allows us to look at the effects of family structure on an array of traits relating to sexual maturity, reproduction, and risk-taking. Our results show that, for both sexes, living with a single mother or mother and stepfather during childhood was often associated with faster progression to life history events and greater propensity for risk-taking behaviours. However, living with a single father or father and stepmother was typically not significantly different to having both natural parents for these outcomes. Our results withstand adjustment for socioeconomic status, age, ethnicity, age at puberty (where applicable), and sibling configuration. While these results support the hypothesis that early family environment influences subsequent reproductive strategy, the different responses to the presence or absence of different parental figures in the household rearing environment suggests that particular family constructions exert independent influences on childhood outcomes. Our results suggest that father-absent households (i.e. single mothers or mothers and stepfathers) are most highly associated with subsequent fast life history progressions, compared with mother-absent households, and those with two natural parents.

## Introduction

Researchers in a wide variety of disciplines have been interested in the influence of family structure on child outcomes, and there is now a great deal of empirical evidence demonstrating that family structure is correlated with particular child outcomes [1], [2]. Notably, families comprised of two natural parents (‘intact’ families) tend to result in slower reproductive development for children, at least in Western contexts where the nuclear family is the normative family structure [3], [4]. A number of hypotheses have been proposed to explain this finding [1], [5]–[7]. Perhaps the simplest and most convincing of these arguments uses parental investment to explain these findings: Ellis [1] suggests that children growing up in families with substantial parental investment benefit from delaying maturity in order to capitalise on this high quality investment. We build on this argument to explore whether and how family structure influences later child outcomes, by assuming that family structure is an indication of the type and quality of parental investment received by a child. We extend previous empirical work by using a large and rich dataset which allows us to compare across five different categories of family structure (intact family, single mother, mother + stepfather, single father, father + stepmother), and to investigate an unusually diverse range of outcomes, including sexual maturation, reproductive behaviour, and risk-taking behaviours.

A key tenet of modern evolutionary theory's principle of inclusive fitness is that parents are favoured by natural selection to allocate investment in their offspring [8]–[10]. Inevitably, there is variation in levels of parental investment received by offspring due to ecological constraints: given limited resources, parents must make trade-offs in order to maximise their own long-term fitness outcomes. Parental differences in resources such as wealth, kin support, and parental experience, are all factors that can mediate the outcomes of these trade-offs and result in variation in the investment received by individual offspring. All else being equal, an evolutionary model of modern human family structure predicts that families comprised of two natural parents will typically result in higher levels of investment received by offspring than families with only one natural parent, since investment is coming from two parents rather than one [11], [12]. Similarly, when parental unions dissolve, the investment received by children is likely to be reduced. Single parent families may therefore, on average, struggle to invest in children at rates equivalent to two-parent families. This deficit in investment may be offset by introducing a surrogate parent into the household. However, stepparent families are not expected to invest as highly in offspring as natural parent families due to lack of shared genetic interests between stepparent and stepchild [13]. Stepchildren will be competitors with any existing or future biological children stepparents may have and thus, from a theoretical approach, on average stepparents are expected to invest less in stepchildren in favour of genetically related children [14], [15]. Introducing a stepparent into the household may also reduce investment from the biological parent in existing children, since they will be diverting some resources from parenting effort into mating effort and their relationship with the new partner [16]. The model we employ here does not necessarily assume that the amount of investment received by a child in a single parent family is exactly that received from the same parent in a dual parent family. This is partly because some ‘parental investment’ may be made up by other family members stepping in to help replace a missing parent, given the cooperative nature of human reproduction [17]; and partly because parents may adjust their level of investment after losing a partner. However, this adjustment may be either increased to compensate for the missing parent, or decreased to invest in mating effort to attract another mate. We do however assume that, at least in contexts where paternal investment is substantial, children who lose a natural parent through death or divorce will overall receive lower levels of parental investment than those who grow up with both natural parents. (The authors want to make clear that we are not advocating that an intact family is necessarily ‘better’ than other family types. We recognise that single or step parents may indeed provide well for their families. This study aims to test an evolutionary hypothesis of genetic relatedness by examining how intact families compare to other family types with regard to offspring's life history course, and carries no value judgement.)

Constraints on parental investment may further depend on the sex of the parent that is missing. The fact that mothers are always certain of their maternity while fathers can never be certain of their paternity, means that mothers should invest more in their children than should fathers (again, all else being equal) [18]. Human mothers initially invest more than fathers do, due to the physiological constraints of pregnancy and breastfeeding on females [19]. After weaning however, paternal investment may become substantial [5], [20]; in wage-labour economies, fathers may be the bread-winners meaning that they probably provide more indirect care by way of resources while mothers are likely to confer more direct care on the child. This sexual division of labour in which fathers provide more indirect care while mothers provide direct care holds across most societies and economies, although in subsistence economies mothers typically also provide considerable resources, in terms of calories, for children [21]. Holding wealth constant (single fathers may be wealthier than single mothers since in wage-labour economies men tend to have more resource-earning power than women), we may expect that mother absence would have a stronger impact on offspring outcomes than would father absence, and that a single mother should be more advantageous for the child compared with other non-intact families due to a larger investment in her children [22].

Within a wage-labour skills-based economic setting, children who receive high levels of parental investment are expected to take slower developmental life history courses compared with less advantaged children [23]–[25]. This is because these high levels of investment can be allocated towards enhancing the child's embodied capital [26], [27], resulting in longer periods spent in education or career development and thereby delaying reproduction. Ellis [1] has proposed that the delayed progress to life history events in children in intact families in such economies results from children from high-investing families capitalising on a high quality developmental environment by extending their childhoods and delaying the onset of puberty. Conversely there is no need for a child who is subject to poor quality parental investment to delay maturity as the benefits to remaining in childhood (prepubescent) are limited. A shortened childhood leads to early pubertal maturity, and so to an earlier progression to sexual and reproductive events. Ellis's hypothesis does not discriminate between maternal or paternal investment, nor does it rely on predicting what the social world will look like in the long term future [1].

The current study aims to develop Ellis's [1] model by examining the effects of different family types in terms of the potential parental investment each one can offer, with regard to offspring's ensuing life history development. We consider that levels of parental investment will vary depending on the specific type of family construction the child is reared in. We are able to do so by examining the differences in life history strategies by comparing children from intact families to those from both types of single parent families and both types of stepparent families, within a single population. Ellis asserts that his model applies only to the timing of life course events and not to other related traits like sociosexuality (e.g. high numbers of sexual partners) and risk-taking behaviour; it predicts only the length of childhood and timing of puberty and reproduction. Given that others have argued that such traits are linked to a fast life history strategy [3], [5], [28], we do not limit our analyses to the timing of life history events but also test whether family structure is linked to sociosexuality and risk-taking behaviours. Based on our simple model of parental investment, as a function of genetic relatedness, we predict that children from intact families will adopt a slower life history strategy compared with those raised by single parents and stepfamilies. Further, stepparent families should produce children with the fastest progressions; tentatively we may predict that natural fathers and stepmothers would promote a faster life course compared to mothers and stepfathers (due to the genetic certainty of mothers). We also expect mother absence to have the strongest impact in accelerating offspring life course development, compared with father absence. Holding wealth constant, our predictions for the family structure with the greatest to the least parental investment are:
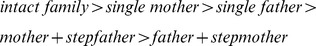



This results in a rather simple hierarchical model based solely on expectations in parental investment based on genetic relatedness. We are aware that this is not the only way one could structure the model as many other factors may come into play, and ‘parental investment’ may be variable even within family types. For example, Nettle and colleagues [29] found that women who were separated from their mothers for short periods during childhood had earlier first births than those who were never separated from mothers, but if that separation was relatively long (for two years or more – possibly indicating the death of the mother), first births no longer differed from women who were not separated. Nevertheless, we use this model as a simple starting point.

Although we have framed our study so far in terms of the differential parental investment children will receive in different family structures, two alternative hypotheses have been proposed for why early family life disruption should predictably have an impact on the timing of life history events. Firstly, Chisholm [5] suggests that unstable childhoods are perceived as cues to increased mortality rates, in which case earlier reproduction is appropriate in order to offset the risk of dying before producing viable offspring [5], [30]. This hypothesis does not discriminate between types of family structure in terms of their influence on child outcomes; rather it predicts that family disruption in general is important as an overall cue of environmental instability, i.e. all non-intact family types should confer equally ‘fast’ outcomes compared to families with both natural parents. A second hypothesis, first proposed by Draper and Harpending [6], is that children who grow up in father-absent households go on to imitate that behaviour by entering into low-commitment relationships themselves. This is because their childhood experience is assumed to be predictive of future options where mates are deemed unreliable [6], [7]. This second hypothesis refers specifically to father absence, as paternal investment is considerably variable between populations and individuals [31], [32], whereas maternal investment is typically high regardless of paternal investment. Father-absent daughters should thus expect that future mates are not likely to be good investors, and sons are likely to grow up to be low investing mates themselves. Conversely, children who grow up in families with their natural father are expected to psychologically gauge that paternal investment (either from their future mates, or as future mates) is likely to be high. In this scenario we would expect to find that father-absent family structures (i.e. single mothers and mothers with stepfathers) have a stronger impact on child outcomes than do mother-absent families.

One problem with both alternative hypotheses, however, is that they assume that current conditions are a good indicator of future conditions later in life, which may not be the case for long-living humans inhabiting unstable environments [33]–[35]. The second model also suggests that father absence should be indicative of male behaviour at the community level, and should predict a relatively stable mating ecology. But if, for example, a girl grows up in a father-absent family yet all her neighbours have intact families, then perhaps her perception will not be that most men are ‘cads’, despite her personal circumstances. The realities of dynamic socioecologies and individual-level effects of father absence may therefore be context dependent. On this point, we note the important caveat that empirical research correlating father-absence with faster life history strategies has been produced almost entirely from industrialised populations. Research outside the industrialised world finds that father absence has more variable effects on children's life history outcomes, suggesting that a simple model of early life disruption leading to faster life history strategy does not apply in all contexts [36]–[38]. The remainder of this introduction outlines in more detail what we might expect from each family type in an industrialised setting.

### Two natural parents

We expect that living with both natural parents should be the most beneficial scenario for children due to the genetic interests of the parents, although variation in resources will influence how much parents can invest. The impact of family structure on children's outcomes has been empirically tested in a large number of studies with fairly consistent findings, at least in high-income settings. In such settings, growing up with both natural parents has been found to promote better educational outcomes [39], better adult mental health [40], and a slower sexual and reproductive pathway in men and women [3], [6], [25], [41]–[43], when compared to other family setups such as living with a single parent or stepparents. What is not yet clear, however, is whether these findings are related to the initial reasons for the change in family structure (e.g. death or divorce), or if they are a product of the influence of acquiring or lacking alloparents [44], specifically stepparents. While we cannot determine the causes of non-intact families in the current study, we are able to examine the costs and benefits to children of living with different alloparents.

### Single mother

Considerable research in low income settings finds that the loss of the mother is typically much more detrimental to child outcomes than the lack of a father, although this literature tends to focus on the health and mortality outcomes of young children rather than their subsequent reproductive behaviour [44], [45]. Similarly, we expect single mothers to most closely represent intact families due to mothers' genetic certainty of maternity and their generally greater levels of investment, compared to other family formations. This prediction only holds however if wealth remains constant, as in high-income settings it is often the case that fathers are contributing parental investment through their higher earnings, which can have a significant impact on children's outcomes.

Empirical evidence finds that children raised in single mother households typically do look rather different from those raised in intact families, for example, in terms of earlier reproductive development [42], [43]. However, the evidence also generally does support the idea that single mother families more closely resemble intact families in high income contexts once socioeconomic status is controlled for, and often finds that single mother families are an advantage to children compared to step families. For example, Biblarz and Raftery [22] found that compared with intact families, children living with single mothers had lower educational attainment and socioeconomic status in later life but that this was accounted for by the mother's socioeconomic status. Having a single father or either type of stepparent had worse consequences for children than did living in a single mother family, after controlling for socioeconomic status. The role of wealth is important here as single mothers may face trade-offs between increasing the household budget, perhaps by working more or remarrying, against the potentially negative consequences this may pose to her children, such as being away from children more, or risks associated with presence of a stepfather.

### Single father

Although we expect somewhat lower parental investment from single fathers than single mothers, we might expect single fathers to be similar to single mothers compared to step-parent families, as although they cannot have complete paternity assurance, cross-culturally non-paternity (of men's putative children) is known to be relatively low – less than 2% [46]. Few studies have examined the impact of single fathers on children, largely because single fatherhood is relatively uncommon. However, using longitudinal U.S. data, Hofferth [47] found that living with a single father was not significantly correlated with children's cognitive abilities but was correlated with an increase in children's behavioural problems, compared to children from two natural parent families. As mentioned in the preceding section, Biblarz and Raftery [22] found that children with single fathers had poorer educational performance than those from single mother or two natural parent families. The current study is able to extend these research findings by testing the influence of single fathers on outcomes related to reproduction and risk-taking.

### Stepparents

We place mothers plus stepfathers ahead of fathers plus stepmothers in our heirarchical model. Most studies that examine the long-term effects of stepparents tend not to differentiate between the types of stepfamilies and argue that marital disruption and familial conflict *per se* is more detrimental than the sex of the outgoing or incoming parental figure. Other studies consider only one or other type of stepfamily and so are not able to directly compare the impact of stepmothers versus stepfathers [3], [48]. If we follow Daly and Wilson's [49] argument of biased genetic interests, we would expect the effects of both male and female stepparents to be the same. Although Daly and Wilson discuss the dangers of stepparents with regard to homicide and maltreatment of stepchildren, from a Darwinian perspective (i.e. genetically), they argue and empirically demonstrate that among those cases of ‘harmful’ stepparents, stepparents of both sexes are equally harmful. We however argue, based on our simple model of parental investment, that while stepfamilies overall may have relatively negative outcomes for children, biological mothers should confer higher investment in children compared with biological fathers (all else being equal). In support of the argument that stepfamily households should differ from intact families, Hofferth [47] found that American children from stepfather families exhibited more behavioural problems than those from intact families. Bogaert [43], however, found no relationship between having a stepfather and age at menarche among contemporary American women once father absence had been controlled for, suggesting that though family disruption due to the loss of a father from the household may influence child development, the addition of a stepfather was not a significant factor.

There are many different contexts which introduce further complexity into these familial and developmental life patterns, including the presence of step siblings [50], the sex of the stepparent-child dyad [51], and the age of the children [52]. For example, Daly and Wilson [13] make clear that stepfamilies are not necessarily a ‘bad’ thing overall. This is because “[s]tep-parents are primarily replacement mates, and only secondarily, replacement parents” [13] p64. It makes sense then that given the incredible amount of investment required to successfully raise a human child [17], a new mate would show solicitude and care towards, or at least tolerate, their new partner's children [14], [15]. Stepparents can be beneficial for stepchildren, although for some outcomes this is sex-dependent, with the greatest benefit found for same-sex stepparent-stepchild dyads. For example, Vaden-Kiernan and colleagues [53] found that urban American boys living with a mother and stepfather showed less aggression at school, compared with those who lived with single mothers, and were no different to those living with both natural parents. Similarly, Clingempeel & Segal [51] found that, in Pennsylvania (U.S.), longer durations of having a stepmother enhanced the stepmother-stepdaughter relationship although if the daughter maintained prolonged contact with her natural mother, her relationship with her stepmother was tempered. The positive relationship between stepmother and stepdaughter was associated with lower inhibition and aggression, and higher self-esteem in daughters. This suggests that a stable maternal figure may compensate daughters, at least to some extent, for the loss of the genetic mother. Here we directly test whether different family structures affect boys and girls differently by analysing each sex separately, and we are able to compare families with both types of stepparent against both intact families and both types of single parent families.

Some research also suggests that stepparental influences vary by context. For example in a comparison of two historical populations, the effects of stepparents appeared to vary in relation to resource availability. Willführ and Gagnon [54] found that stepfathers were beneficial for child survival in historical Quebec (Canada) but there was no effect of stepmothers, while in Krummhörn (Germany), stepmothers reduced child survival but there was no effect of stepfathers [54]. They attribute these differences between populations to differences in resource availability they each faced; in Quebec, resources (in this case, land) was plentiful and could support high fertility levels, so stepmothers were at an advantage and were able to produce more offspring. These offspring had no incentive to compete with stepsiblings as there was little resource stress. In Krummhörn, land was very scarce and so stepmothers would favour future offspring at the expense of their stepchildren's survival. In other historical populations, stepmothers and stepfathers have both been found to be beneficial for offspring survival, for example in historical Sweden, perhaps because of the ‘social network’ advantage a two-guardian family bestowed [55], [56].

### Context-dependent variation

Research on the effects of family composition on children's reproductive outcomes later in life is prevalent in post-demographic transition contexts but little is known about these relationships within higher fertility settings. The existing literature in such settings on family structure and child outcomes is mostly restricted to examining the impact of family structure on the survival and health of young children, as described above for stepparents in historical populations. Work on family structure and children's reproductive outcomes is primarily concerned with the role of fathers, and is focussed on the roles that fathers play in their children's lives, rather than assuming that parental absence is simply a cue for environmental instability. Effectively it largely assumes, as we do here, that parental absence is an indicator of lower parental investment within a family. For example, for men, father absence tends to delay progression to reproductive life events which is due variously to fathers' roles, such as in arranging initiation practices among the Australian Martu [57] or providing the financial assistance necessary to marry among the Belizean Maya [58]. Similarly, fathers may be important for arranging marriages, in which case the absence of a father may delay reproductive events for women too, as in the Gambia [37]. Alternatively, in a transitional setting, rural Trinidad, where sexual purity is considered a virtue, fathers act as guards of their daughters' reputations. This means that girls who grow up without their natural fathers will tend to marry younger [59]. These findings indicate that fathers provide practical and material help for children in pre-demographic transition contexts. While it is hard to distinguish between different hypotheses with correlational analyses, if we find that father absence delays sexual or reproductive events, this might be an indication that fathers are performing some role that accelerates their children's entry into reproductive behaviour in this historical U.S. population.

### Risk-taking and sex differences

The child outcomes that we are interested in here include a number of variables denoting the timing of sexual maturity and reproductive behaviour. We are also interested in outcomes related to risk-taking behaviour. Risk-taking variables are essentially measurements of high propensities for activities that may be detrimental to one's health, social well-being, and financial security. Risk-taking may be associated with the timing of life history events because both high-levels of risk-taking and early reproductive behaviour may be driven by a ‘future discounting’ strategy [28], [60]. Further, risk-taking itself increases the chance of premature death due to, for example, unintentional accidents or poor health [49], which may lead to increased future discounting. For men, high levels of risk-taking is understood to be a high-mating, low-parenting strategy [61], [62]. Non-sexual risky behaviour may constitute display-type behaviours associated with costly signalling in order to obtain mates, while sexual risk-taking can be seen as a direct attempt to increase low-cost fertility. As a putative proximate mechanism, financial risk-taking has been correlated with elevated levels of testosterone [63], which in turn is associated with increased mating effort [64].

Although there is little reason to expect that the antecedents to risk-taking should be different in women compared to men, less is known about female risk-taking, except that on average women tend to exhibit less risky behaviour than men do [65]. Belsky and colleagues [66] found that maternal harshness (a proxy for unstable family life), by way of early pubertal maturation (in girls), promoted sexual risk-taking behaviour but not other types of risk-taking. Other studies have found that early puberty is related to higher levels of both sexual and non-sexual risk-taking in both boys and girls [67], and that in some cases both types of risk are themselves correlated [68].

More broadly, there might be some reason to expect that male children would be more sensitive to familial environments compared with female children, because boys are generally more costly to produce and need more energy in order to grow [69], [70]. Among infants, boys also tend to die more easily than girls [71]. Although this argument generally pertains to very young children, it is possible that this sensitivity continues in later life, making boys more susceptible to influences from the social environment throughout childhood. One experimental study which primed respondents to mortality cues found that this was associated with a desire for higher numbers of children than those not primed, but only for men [72]. It may be that because males are more easily able to adjust their reproductive strategies compared with females who incur much higher compulsory costs to childbearing, young males are more responsive to environmental cues. We may therefore expect male children to have higher proportions of statistically significant effects associated with faster life history trajectories than female children.

### The current study

Clearly, there are many complex, dynamic, ecologically contextualised issues that need to be considered when trying to isolate the different, and concomitant, influences that certain family structures can have on children over the long-term. In the present study, we draw upon a rich data source that allows us to take at least some of these factors into consideration. We are able to test if there are different effects depending on which particular familial composition the child lived with: both natural parents, single mother, single father, mother and stepfather, and father and stepmother. By comparing outcomes from these different family configurations within the same study, we can potentially assess which family types constitute higher or lower levels of parental investment, compared with intact families. This also enables us to verify whether generally low parental investment is more important than specific family structures, in which case we should find no difference in whether the child was raised by either a single parent or a stepfamily. Finally, we can examine if there are different patterns of results between the sexes.

## Methods

### Data

We use data from the Original Kinsey Survey collected from 1938 to 1963 in the United States by the then named Institute for Sex Research at Indiana University. Interview methodology and initial reports from this survey were published in ‘The Kinsey Reports’, two historically important books on human sexual behaviour [73], [74]. This is an unusually rich historical dataset in that respondents were interviewed for several hours about very detailed aspects of their sexual behaviour [75]. Comprehensive information on demographic, socioeconomic, childhood family structure, education, and health were also collected. Contemporary large-scale surveys tend to collect this sort of demographic information but with rather limited data on later outcomes; for example, often only ages at puberty and births are recorded and little about other sexual behaviours which may lead to a first birth. Such data are also commonly collected only for women. The Kinsey dataset allows us to examine a number of different childhood family situations in relation to a comprehensive set of variables constituting sexual behaviour, as well as marital and reproductive outcomes, within a single U.S. population. The majority of studies that have looked at the relationship between early life conditions and sexual maturity or reproductive outcomes tend to focus on one or two outcome variables only. Due to data restrictions it is often impossible to do more than this. Yet milestones like puberty, first sex, marriage, and first births are often assumed to be different facets of a single reproductive strategy, either fast or slow, and so investigating effects on one of these outcomes is taken to represent the other non-measured outcomes. By examining correlations between family structure and all of these outcomes, we are able to indirectly test whether it is safe to assume that early life conditions affect all life history outcomes equally.

Alfred Kinsey and his team endeavoured to amass a large enough sample in order to draw general conclusions about human sexual behaviour and to compensate for the deliberate sampling bias. He avoided using a random sampling technique as he believed that the nature of the interview topics would lead to an untenable number of refusals [73]–[75]. Instead, Kinsey and colleagues chose to interview people on the basis that they were willing to participate. Kinsey and his staff were able to collect around 18,000 in-depth interviews until 1963, providing a rich, comprehensive sample of participants from many diverse backgrounds. The current study analyses only include results from participants who were aged 18 years or older at the time of the interview (91.5% of the original sample), leaving us with a study sample comprising 9,039 men and 7,168 women.

### Study design

#### Primary Variables

Our principal independent variable of interest is *family living situation from age six to eight (inclusive)*. We chose this particular age as this it is often considered that up to around age five to seven years is the child's critical developmental period [6], [7], [60]. This variable is divided into eight categories: intact family (reference category), single father, single mother, father and stepmother, mother and stepfather, foster home (non-relatives), foster home (relatives), or an institution (e.g. an orphanage). We are primarily concerned with the outcomes pertaining to the first five categories and so only these are discussed here (but see [24] for a comparison of kin fostered and non-kin fostered children). A breakdown of these categories by sex is shown in Table 1. Our dependent variables are: age at puberty, age at first petting, ever had premarital sex, number of sexual partners, progression to first sex, engaged in extra-marital sex, progression to first marriage, more than one marriage, age at first birth, gambling (more than a little), and use of illegal drugs. We used linear or logistic regression analyses to model our data for those dependent variables where full information on all participants was known; we used discrete-time event history analysis for those outcome variables where not all individuals had experienced the event in question (to enable inclusion of censored cases). Descriptive statistics of our dependent variables, and the modelling techniques used for each, are presented in Table 2.

**Table 1:** Family breakdown by sex.

	MEN	WOMEN	TOTAL
INTACT FAMILY	7,227	5,857	13,084
SINGLE FATHER	166	116	282
FATHER + STEPMOTHER	133	148	281
SINGLE MOTHER	669	555	1,224
MOTHER + STEPFATHER	271	189	460
**TOTAL**	**8,466**	**6,865**	**15,331**

Distribution of family situations by sex (foster families and institutions omitted).

**Table 2:** Descriptions of dependent variables.

Dependent variables	WOMEN			MEN			Model type
Continuous:	n	Median (years)	range	n	Median (years)	range	
Age at puberty	4695	12	8–21	4918	13.3	6–22	Linear regression
Age at first petting	6812	16	9–42	8664	15	7–44	Linear regression
Age at first sex^1^	7163	21	10–35	7369	18	10–35	Event history
Age at first marriage^1^	7153	24	12–48	9038	27	13–48	Event history
Age at first birth^2^	1270	24	12–43	1762	26.5	13–54	Linear regression

Categorical:	n (%)						
Premarital sex	**Yes:** 3121 (64.8)	**No:** 1695 (35.2)		**Yes:** 6069 (86.4)	**No:** 954 (13.6)		Binary logistic
1+ marriages	**Yes:** 705 (19.6)	**No:** 2885 (80.4)		**Yes:** 783 (18.7)	**No:** 3401 (81.3)		Binary logistic
Illegal drugs	**Yes:** 261 (3.8)	**No:** 6454 (96.2)		**Yes:** 928 (10.3)	**No:** 8111 (89.7)		Binary logistic
No. of premarital sex	**0:**	**1–5:**	**6+:**	**0:**	**1–10:**	**11+:**	Multinomial
partners	1695 (35.2)	2477 (51.4)	644 (13.4)	954 (14.7)	3845 (59.1)	2224 (34.2)	Logistic
Gambling	**None:**	**Little:**	**More:**	**None:**	**Little:**	**More:**	Multinomial
	4905 (72)	1419 (21)	478 (7)	3737 (49.4)	2119 (28)	1709 (22.6)	logistic

1 These medians were calculated including censored cases.

2 Age at first birth is for married respondents and only for those who were age 30 or over at the time of interview.

Age at puberty was derived by calculating an average of three puberty markers available in the dataset. For women, these were: age at first menses, age at onset of breast development, and age at onset of pubic hair, in years. We also conducted a more sophisticated index using principal components analysis, but because both methods produced similar results in the models we retained the simpler index for ease of interpretation. For men the same procedure was conducted but using different component variables: age at voice-breaking, age at first ejaculation, and age at onset of pubic hair, also in years. We analysed the individual puberty markers separately to test for any particular differences between them, but the results remained largely the same as when we used the compound variable for puberty.

In order to measure precocious sexual behaviour we used two variables: age at first petting, and age at first sex. Petting is described by the survey interview as “…hugging, kissing or petting – anything more than a goodnight kiss?” Gebhard & Johnson [75] p248. As 96.3% of participants had already experienced petting, this was analysed using a linear regression model, using only those participants who had already experienced the event. For age at first sex, however, 24% of the sample had not yet had sex, so we used a discrete-time event history analysis to model this outcome in order to accommodate those censored cases [76]. We modelled progression to first sex between ages 10 and 35 years since few individuals experienced first sex before or after these ages. We also looked at progression to marriage (where marriage included having lived with a partner as a common-law spouse for one year or longer), and age at first birth. We modelled progression to marriage as a discrete-time event history model in order to incorporate censored cases, as only half had been married by this time. We analysed the probability of marriage from age 12 to the age of 30 years, as 95% of those married were married by then. Age at first birth was modelled using a linear regression analysis and included only married respondents who had a birth as we only have data on births for married respondents (although most births occurred within marriage during this time period [77]). We limited this analysis to those women who were aged 30 years or older at the time of interview to reduce any bias towards women who had first births relatively young. We also examined total number of marriages, as we assume this is an indicator of a low-commitment strategy. Here, number of marriages refers only to ‘legal’ marriages because the interview question was framed this way. As less than half of the sample was ever married at the time of the interview and only 9% of the total sample had more than one marriage, we excluded never-married individuals for this analysis and modelled this as a binary outcome with ‘married once’ or ‘more than once’ as the two outcome possibilities.

We constructed two variables derived from the same survey question to measure sexual activity. Firstly, whether or not the individual had engaged in any premarital sex, and secondly, if they had, with how many partners. Both of these variables are meant to represent some degree of sociosexuality. On average, having many sex partners is indicative of relatively low levels of commitment, possibly indicating more of a mating rather than a parenting strategy [5]. Relatively few respondents reported premarital sex, making it therefore plausible that having had even one premarital sex partner makes one qualitatively different from those who had none. Our second outcome was having had more than five premarital sexual partners, for women, and more than ten sexual partners, for men. Although these are a somewhat arbitrary numbers, by examining the distribution of data for both men and women, we consider that this variable captures people who had relatively large numbers of sex partners in comparison with the rest of the sample. We modelled this outcome using a multinomial logistic regression, but only present the point estimate for having ‘many’ sex partners in our results section (where the reference category is no premarital sexual partners).

To examine both sexual and non-sexual risk-taking activities, we derived a sexual risk-taking variable which coded for whether a person engaged in extra-marital sex, which was modelled using a binary logistic where 1 = ever had and 0 = never had. Extra-marital sex may be construed as risky behaviour as the individual stands to lose their spouse, children, and financial security should they be found out. Non-sexual risk-taking was operationalised as a propensity for more than a little gambling, and for the use of illegal drugs. These activities may confer short-term benefits but also pose obvious financial and health costs. The dataset included a 3-category variable comprising ‘no gambling’, ‘only a little’, ‘more than a little’. We consider only the last category as risk-taking behaviour because only that category involved gambling for high financial stakes. We used a multinomial logistic regression to analyse this variable but only present the odds ratio for gambling ‘more than a little’ in the results section (where the reference category is ‘no gambling’). Finally, we derived a binary variable for illegal drug use (any use or none) because not many people in the dataset reported using illegal drugs at all and modelled this with a binary logistic regression.

#### Key Control Variables

In all models we adjusted for socioeconomic status, recorded as parental financial situation from age 14 to 17 years (the only time period for which wealth data in childhood is available), because there is a known link between wealth and life history strategy [78], [79]. Lack of financial resources can be considered a cue to a poor early environment, resulting in a faster life history progressions [80]. We further controlled for number of siblings because this has been shown to affect reproductive outcomes in other studies [42], although the sex of siblings and whether they are younger or older often makes a difference [81], [82]. Our number of siblings variable includes all those children raised in a household with the respondent, as respondents were not asked to distinguish between full, half, or step siblings. Finally, because age at puberty is found to be associated with age at various sexual activities [83], reproductive outcomes [84], and engaging in both sexual and non-sexual risk-taking behaviours [67], we controlled for age at puberty in the relevant models, i.e. all except for the ones with age at puberty as an outcome. It is possible that puberty could be seen as a mediating effect of family structure on these outcomes and we want to avoid the possibility that any correlations we see between family structure and later outcomes are due merely to associations between family structure and age at puberty.

#### Other Controls

We further controlled for ethnicity (15,048 of our sample were identified as white and 2,161 as non-white), year of birth (which ranged from 1848 to 1941), birth order, and birth order squared (due to the non-linear nature of birth order effects). In the discrete-time event history models we were able to control for respondent's age at interview as well as birth cohort.

## Results

We present results for all 22 models (11 for each sex) with outcomes denoting various aspects of life history in Table 3. We only include the family composition categories of interest here and the key controls of puberty, SES and family size, which we also discuss. All models also adjust for ethnicity, respondent's age at interview, birth cohort, birth order and birth order squared, and for the event history models only, time and time squared (which was measured in years from the age at which the respondent was first exposed to the risk of the event happening: age 10 years for the progression to first sex model and age 12 years for marriage); point estimates for these are not shown here. The full models showing all effects, including those of other types of childhood family situation (foster care and institutions), as well as results for all control variables, are available in Tables S1a (women) and S1b (men) of the supplementary material. Note that the type of point estimate differs across models: age at puberty, first petting, and first birth show beta coefficients and the rest show odds ratios, with the exception of number of sex partners and propensity for gambling, which are relative risk ratios. Event history model odds ratios are interpreted as the likelihood of an event occurring at each year, given that the event has not yet occurred. Note that negative coefficients presented in the linear regression models, and estimates more than 1 for the logistic and event history models should be interpreted as accelerating effects.

**Table 3:** Results for all outcomes in relation to family structure.

	MOTHER ABSENCE		FATHER ABSENCE		KEY CONTROL VARIABLES		
	SINGLE FATHER	FATHER & STEPMOTHER	SINGLE MOTHER	MOTHER & STEPFATHER	LATER PUBERTY	HIGH SES	LARGE FAMILY SIZE
**WOMEN**							
AGE AT PUBERTY^1^ (n = 4624)	0.06 n.s.	−0.04 n.s.	−0.03 n.s.	−0.07 n.s.	n/a	−0.05***	0.11***
AGE 1ST PETTING^1^ (n = 4413)	−0.22 n.s.	−0.16 n.s.	−0.24 n.s.	−0.65**	0.33***	0.15**	0.02 n.s.
AGE 1ST SEX^2^ (n = 4622)	1.87***	1.31*	6.13***	6.25***	0.98 n.s.	0.82***	1.08***
PREMARITAL SEX^2^ (n = 2992)	1.41 n.s.	0.80 n.s.	1.44**	1.05 n.s.	0.95 n.s.	1.01 n.s.	1.02 n.s.
6+ SEX PARTNERS^3^ (n = 2992)	1.61 n.s.	0.94 n.s.	1.62*	1.20 n.s.	0.97 n.s.	0.91*	1.07§
AGE AT MARRIAGE^2^ (n = 4612)	1.46*	1.30 n.s.	1.10 n.s.	1.69**	0.98 n.s.	0.83***	1.08***
2+ MARRIAGES^2^ (n = 2193)	1.01 n.s.	1.22 n.s.	1.18 n.s.	1.52 n.s.	0.91*	0.86***	1.01 n.s.
AGE 1ST BIRTH^1^ (n = 1024)	−34.92*	1.93 n.s.	−9.81 n.s.	−49.97**	2.53 n.s.	7.48***	−3.63**
EXTRA-MARITAL SEX^2^ (n = 2249)	0.93 n.s.	0.58 n.s.	1.01 n.s.	1.08 n.s.	1.01 n.s.	1.04 n.s.	0.96 n.s.
GAMBLING^3^ (n = 4464)	1.42 n.s.	0.53 n.s.	1.27 n.s.	2.22**	0.96 n.s.	0.93 n.s.	0.97 n.s.
ILLEGAL DRUGS^2^ (n = 4422)	1.35 n.s.	1.67 n.s.	1.62*	2.69**	1.03 n.s.	0.74***	0.87*
**MEN**							
AGE AT PUBERTY^1^ (n = 4749)	0.31*	0.35*	0.12 n.s.	0.18 n.s.	n/a	−0.06***	0.05***
AGE 1ST PETTING^1^ (n = 4582)	−0.20 n.s.	−0.24 n.s.	−0.16 n.s.	−0.29 n.s.	0.54***	0.03 n.s.	−0.05*
AGE 1ST SEX^2^ (n = 4744)	1.29*	1.08 n.s.	0.21 n.s.	1.28*	0.95***	0.91***	1.07***
PREMARITAL SEX^2^ (n = 3654)	1.19 n.s.	1.09 n.s.	1.43 n.s.	2.81*	0.99 n.s.	0.95 n.s.	1.14***
11+ SEX PARTNERS^3^ (n = 3654)	1.67 n.s.	0.83 n.s.	1.61§	3.48*	1.00 n.s.	0.89**	1.17***
AGE AT MARRIAGE^2^ (n = 4748)	1.10 n.s.	1.01 n.s.	1.01 n.s.	1.18 n.s.	0.94**	0.96*	1.03 n.s.
2+ MARRIAGES^2^ (n = 1996)	1.62 n.s.	0.21 n.s.	1.71**	2.73**	0.98 n.s.	0.88**	1.05 n.s.
AGE 1ST BIRTH^1^ (n = 1128)	13.97 n.s.	6.65 n.s.	−20.04*	−19.28 n.s.	0.08 n.s.	5.60***	−4.76***
EXTRA-MARITAL SEX^2^ (n = 2086)	0.78 n.s.	1.19 n.s.	1.54*	1.03 n.s.	0.92*	0.92**	1.03 n.s.
GAMBLING^3^ (n = 4031)	2.15**	1.08 n.s.	1.64**	1.61*	1.01 n.s.	0.96 n.s.	1.03 n.s.
ILLEGAL DRUGS^2^ (n = 4749)	1.68 n.s.	1.15 n.s.	1.61**	2.55***	0.93§	0.79***	0.96 n.s.

All models also control for birth order, birth order squared, ethnicity, and age. Event history models further control for birth cohort, time and time squared.

note: age at first birth is measured in months, for married people who had a child.

reference category: intact family.

1 beta coefficients.

2 odds ratios.

3 relative risk ratios.

***p<0.001,

**p<0.01,

*p<0.05,

§ p<0.06.

**Figure 1:**
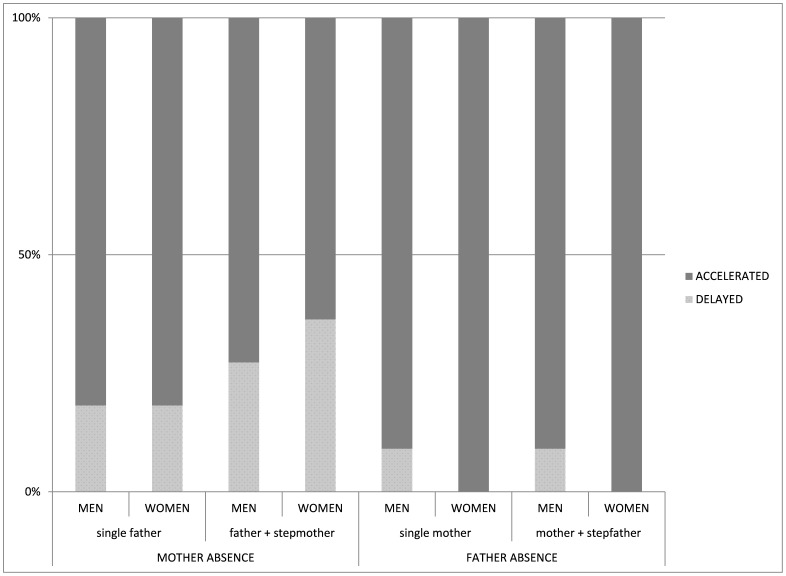
Distribution of models by delayed or accelerated outcomes of events.

**Figure 2:**
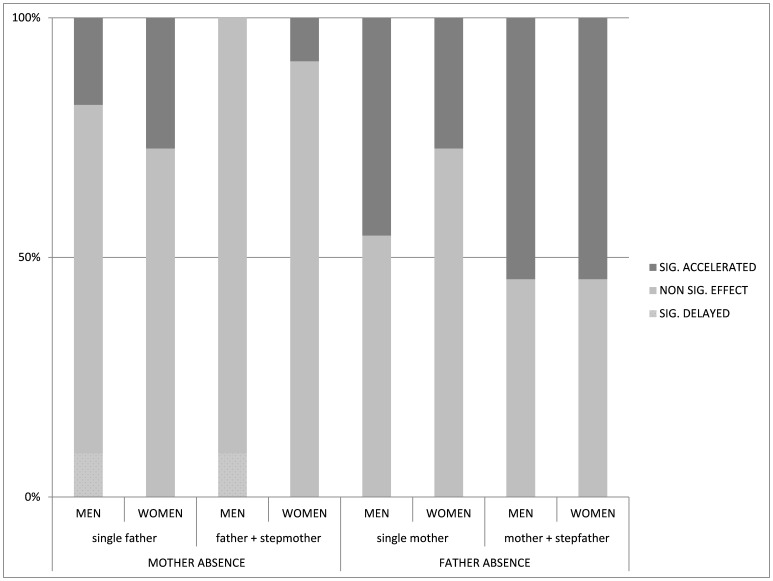
Distribution of models by statistically significant delayed or accelerated outcomes of events, and non-significant effects (i.e. no effect).

Having so many individual models makes it cumbersome to discuss each one separately, in particular because we are ultimately interested in overall patterns of effects to see if there are any general differences between the four types of family formations on these outcomes. We draw attention to the overall patterns that we find in Figures 1 and 2. Figure 1 shows the percentage of models in which negative (i.e. associated with accelerated life history) and positive (associated with slower development) correlations were found for each family type, for both sexes and regardless of significance level. We also indicate the 50% line which is roughly where the distribution of delayed and accelerated outcomes should lie were there to be no effect of family structure on our life history outcomes. It is clear from Figure 1 that all family types have a larger percentage of accelerated life history outcomes than delayed ones, compared to intact families, substantially so in the case of most family structures. This supports previous research in high income settings showing accelerated life histories for those children raised in non-intact families, and is in line with our own predictions. What is not in line with the predictions from our parental investment model is that for father-absent families there are higher proportions of accelerated effects compared with the mother-absent categories, for both men and women.

Figure 2 shows the proportions of our models split into significantly accelerated and significantly delayed outcomes, as well as non-significant effects. While it is clear that many of our models do not show significant differences in life history outcomes between children raised in different family structures, the same broad interpretations can be drawn: (1) non-intact family structures are more likely to be associated with accelerated than delayed life histories; (2) there is a much higher proportion of significantly accelerated associations in the father-absent categories compared to the mother-absent groups (here, particularly in the case of mothers and stepfathers) and (3) the trends are similar for men and women.

Our results show that, broadly, most kinds of non-intact family structure tend to accelerate reproductive and risk-taking behaviour for both sexes, though this is not wholly consistent across all outcomes, family types or the sexes. We find a noteworthy similarity among two broader categories of family construct: father-absent and mother-absent families. Our fast life history strategy findings are strongest for the two father absence categories: for women, all point estimates suggest accelerated behaviour for single mother families (four of which are significant) as well as for mother and stepfather (six of which are significant). For the mother absence categories, nine out of eleven point estimates signify a faster strategy (three are significant) for single father families, while for father and stepmother seven of the eleven models suggest accelerated behaviour (and only one is significant). Progression to first sex includes interaction terms, so while the significance level and direction of the effect remain consistent, to interpret the sizes of the effects one must incorporate the point estimates of the relevant interactions. These are shown in Tables S1a (women) and S1b (men). For men, we see a similar pattern with father-absent family formations showing the highest proportion of statistically significant accelerating associations. For single father families, nine out of eleven models suggest a faster life history (two of which are significant), and for father and stepmother families eight out of eleven estimates denote accelerated behaviour (none significant). For the mother-absent families, single mothers are associated with faster outcomes in ten out of the eleven models (five significant), and in mother and stepfather families, ten of the eleven models suggest a faster life history pace (six significant). The only models which suggest statistically significant delays in life history outcomes are the puberty models for the two mother-absent categories for men: in both single father and father plus stepmother families, boys experience later puberty compared to those in intact families. We elaborate on these results below, focusing on overall patterns of behaviour rather than individual models.

### Family structure

If different family structures simply denote variation in levels of overall parental investment received by children, we proposed the following simple, genetics-based, hierarchical model:
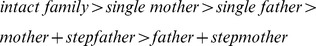
The findings from this study do not support this model. Instead, we find that single fathers and in particular, fathers who are remarried, most closely resemble intact families, and that this pattern holds for both sexes. We found that overall, single mothers, and perhaps especially mothers who remarried, had the strongest impact on hastening the life course of both boys and girls in this sample, compared with intact families. What our results show, for both sexes, is this structure:
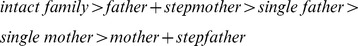



Figures 1 and 2 illustrate this arrangement; it is plain that the proportions of negative associations increase as the family type moves from father and stepmother in the most positive case, to mother and stepfather at the other extreme. While the results for each outcome and for each sex are not wholly consistent across all family configurations, the general pattern that we find overwhelmingly favours grouping our families into mother absence and father absence categories as the most useful way to interpret these findings. The consistent direction of the correlations between both father-absent family structures and female outcomes may lend some support to the idea that our outcome variables constitute a suite of life history variables, in this population at least, which are similarly affected by early life conditions. However, the correlations were not completely consistent in their directions for male outcomes, nor for female outcomes in the mother-absent family structures, which suggests that more direct tests of whether these outcomes can be considered all part of the same life history strategy should be conducted.

### Sex differences and puberty

Interestingly, the overall pattern of our results shows no marked sex differences, although we do find a few sex differences within individual models. The most noticeable sex difference was found when we examined age at puberty. As an outcome, girls' age at puberty was not significantly affected by any particular family type, while for boys, living in either situation from the mother-absent group showed a *delayed* age at puberty. This was the only significant result we found where a non-intact family structure was associated with a slowed down life history event, compared with an intact family. Furthermore, in the other models where we included age at puberty as a control variable, we see that puberty seems to be a more important correlate of later outcomes for men than women: for men, age at puberty is significantly associated with six out of ten behavioural outcomes while for women, there are only two models where puberty was correlated with later outcomes.

## Discussion

Our findings from this historical population in the United States partly match what we see from modern high income, low fertility settings, where low parental investment is associated with numerous outcomes accompanying a faster life history progressions [52], [85], [86]. However, our results vary by the particular type of family context. These findings reflect the general pattern that father absence, for both sexes, promotes a faster life history compared to mother-absent households. This indicates that there is something about the specific family make-up; single mothers and mothers plus stepfathers in particular (i.e. father absence), that affects children over and above general childhood instability. Note however that not all models show significantly different results from intact families, even for the father absence models, so we do interpret our results with some caution. We also find that while the behavioural and fertility markers that we use show some consistency between the sexes, the puberty models show an interesting disparity. Mother-absent boys showed a later age at puberty while we found no significant effect for father-absent boys (although the direction of the point estimates suggest a similar delay), or for any family type and girls' age at puberty (the point estimates were close to zero, but 3 out of 4 suggested an acceleration rather than delay). On the whole, however, we found very few sex differences in the overall patterns of outcomes we tested. We discuss these findings in more detail below in relation to specific hypotheses.

### Intact family > single mother > single father > mother + stepfather > father + stepmother

Firstly, we expected that all non-intact family formations should be associated with a faster life history strategy compared with intact families, but we found little support for this. The majority of the effects from the two mother-absent family types were not significantly different from intact families, indicating that only certain types of non-intact families may pose a problem, in this case, father-absent families. This challenges the hypothesis put forward by Chisholm [5] which would expect that any type of disrupted childhood is an indicator of increased mortality rates, and so would lead to faster life history progressions. We then predicted that single mothers and single fathers would be more like intact families than stepparent families, holding wealth constant. Our findings in this study do not support this prediction. We found that living with a father and stepmother most closely resembled an intact family, followed by living with a single father. Single mother families and those with a stepfather were most frequently associated with accelerated outcomes, compared to intact families. These patterns indicate that, in general, mother absence is more similar to having an intact family than is father absence, and that this holds for both sexes. The structure our findings support is:
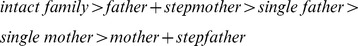



This hierarchical structure lends some support to Draper and Harpending's [6] argument that paternal investment is particularly important for child outcomes and that children from father-absent homes tend to embark on a faster life course, for children of both sexes. Their model specifies that fathers are more important than mothers in terms of a cue to the future social environments. In contexts where relatively little support from others is available, it is also plausible that single mothers are more of a burden to children than single fathers are, as the child might feel an obligation to emotionally support the mother. Although we do not have any direct information about the mothers of the participants in our sample, it is also possible that single mothers are pursuing a faster life history strategy themselves, which may be inherited by the children leading them to follow a similar strategy. This would explain why the remarriage of the single mother does not buffer the child from such a strategy [6]. This argument assumes that early life conditions are predictive of the future and thus that father absence is relatively normative in the local environment that the child finds itself. We are unable to gauge this in the current study however, so we remain cautious about whether our findings really do support this hypothesis.

Ellis's [1] child development hypothesis predicts that low parental investment leads to a shortened childhood with early resultant puberty leading to early onset of other reproduction-related events. We do not have information about actual parental investment but predicted that families with the natural mother would display higher levels of investment given that on the whole human mothers typically invest more in children than do fathers. Our findings do not support this model. We find that father-orientated families are closest to intact families, suggesting that it is these families that confer the highest investment. It is possible that our argument regarding greater maternal investment is flawed and that perhaps there is reason to expect father absence to have a stronger impact than mother absence. One possible explanation for this is that mother absence is relatively rare (in the U.S., children generally live with the mother after marital dissolution [87]) and that single fathers more quickly find alternative allocare which buffers the effect of mother absence, perhaps precisely because fathers and potential allocarers (such as other family members) perceive that the loss of the mother can be extremely detrimental to children. A more important problem for the Ellis model is that we found that no non-intact family type predicted accelerated age at puberty. There are no significant differences for girls, and for boys we find that the two mother-absent family types were associated with *delayed* puberty. However, as Ellis [1] does acknowledge, such a model of low parental investment accelerating life history applies only under conditions of reasonably good nutrition and stable environments, which may not necessarily apply to this historical population. We continue this point in the section on family structure and puberty below.

### Life history as a cohesive strategy

Both the Draper and Harpending [6] and Chisholm [5] models, but not Ellis's [1] model, assumes that early life disruption is correlated not just with the timing of reproduction-oriented events but with other life history traits, such as sociosexuality. Although we cannot directly test whether all our life history outcomes are correlated as part of an overall life history strategy, we would expect consistent effects across outcome variables if this were the case. Although our results are not completely consistent, we do find that the direction of our correlations suggests that family disruption does increase sociosexuality and risk-taking behaviour, as well as accelerating the timing of life history events, which may be interpreted as supporting the hypothesis that these outcomes form a coherent suite of traits. Given, however, that the most notable inconsistent result was the delaying effects of non-intact family structure on boys' puberty – which is considered a key variable in most models which link family structure to later reproductive outcomes – we are hesitant to draw strong conclusions on this point. To somewhat test for interrelationships between life history traits, we tested whether there were associations between age at puberty and our behavioural life history traits. We found that our models lend some support to the hypothesis that earlier puberty would be correlated with accelerated behavioural life history outcomes and greater propensity for risk-taking behaviour, particularly for men. However, this support was not particularly strong, as few of our correlations (particularly for women) were significant, and not all correlations were in the expected direction. And it is still unclear how family formations are actually affecting age at puberty (we discuss this further below).

### Father absence and household wealth

The findings of our study indicate that father-absent family types are the most dissimilar to living with both natural parents, suggesting that father absence has a stronger effect on child life history outcomes than mother absence. As we emphasised in the introduction, paternal investment is often indirect and involves the provision of some kind of resources to children. While we appreciate that this is not trivial parental investment, we nevertheless control for wealth in our models as there is variation in wealth between families, independent of whether or not there is a father present. It is possible that controlling for wealth disguises the parental investment effects of fathers, though this would only exacerbate the difference between mother-absent and father-absent families that we find here. Our results show a number of significant effects of father absence, as well as significant effects of wealth, so we are therefore able to conclude that fathers are important over and above the wealth they may provide. It would be useful to directly test how father absence relates to household wealth by determining how household wealth changes before and after a father disappears from the household; unfortunately such longitudinal data on household wealth are not available in this dataset.

### Stepmothers versus stepfathers

We predicted that mother plus stepfather families would more closely resemble intact families than father plus stepmother, but found quite the opposite: father plus stepmother families were in fact most like intact families of all our family structures, whereas mother plus stepfather was the least like intact families. These results do, however, support previous findings that demonstrate that stepfathers play an important, albeit negative on average, role in stepdaughters' developmental outcomes [3], [88]. We show a similar correlation here for stepfathers and stepsons. For boys, stepfathers may influence them in ways that promote regulation of mating effort over parenting effort, possibly due to competition between unrelated males [89]; if stepfathers feel threatened they may induce highly stressed family environments. It is also possible that a new male in the household diminishes maternal care towards children, which could also increase their stress.

Although we know much less about the effects of stepmothers, at least in terms of reproductive outcomes, our results suggest a positive impact of single fathers remarrying, as the behavioural outcomes of those who grow up in father and stepmother families are largely similar to those in intact families. Note that it is possible that this finding may arise for reasons other than the behaviour of the stepmother. As we suggest above, it may be that when a mother becomes absent, the lack of maternal investment is recognised to be such a problem for the child that she is quickly replaced by other helpers (alloparents). These differing effects of stepmother and stepfather households may also shed some light on the question of whether introducing a stepparent, or if marital dissolution, is the primary factor driving any later outcomes [44]. If marital discord is an innate problem then either type of stepfamily should exert a similar influence on later outcomes. However, we found that stepfathers result in many more significant associations than stepmothers do.

### Family structure and puberty

It is interesting that, unlike what is commonly reported in low fertility settings, we did not find an association between father absence and age at puberty for women. There are not many studies that have looked at family structure and age at puberty in low or middle income settings, although in Malaysia there was also no significant association found between father absence and girls' age at puberty [38]. It is important to note again that this data was collected between the years 1938 to 1963 in the United States, meaning that many of the participants were children during the late 1800s to the early-mid 1900s. It is likely that, in a historical setting such as this, nutritional factors exerted a stronger influence on physiological development than did psychosocial factors. This idea is further supported by the fact that we found a negative association between age at puberty and socioeconomic status for both sexes (see Table 3). That is to say, children from wealthier households began puberty at younger ages than their less financially fortunate peers. The association we find between large sibships and later puberty also supports this argument (see Table 3). It is possible that children from large families compete with siblings for resources and are thus unable to mature as quickly as those from smaller families; this would be consistent with literature on mammalian litter size, correlating larger litters with slower growth and development [90]. One study has demonstrated that it is the specific type of sibling composition that matters [82]. Milne and Judge [82] found that, among contemporary Australian women, presence of older brothers delayed puberty while presence of sisters and younger siblings did not have an effect. They attribute this to the possibility that older brothers are more heavily invested in than daughters are, given that human male children are more costly to produce [91]. This would leave sisters with fewer resources although it is unclear then why there was no effect of younger brothers. We considered this hypothesis by splitting our family size variable by sex and birth order of siblings but found no difference in the effects for any type of sibling composition for women, i.e. all types of sibling exerted a significant positive influence on age at puberty. For men, when the model is run this way there is a positive significant effect of older sisters, younger brothers, and a marginally significant for younger sisters (p = 0.058), but no significant effect of older brothers. This is difficult to interpret but seems to suggest that larger sibships are in general more important than specific sibling compositions.

For men we found that mother-absent households were associated with delayed age at puberty but no significant associations for father-absent households. We might therefore conclude that there is something about living with a natural father that allows boys to extend their childhoods and slow their pace of physical maturity. In another study, using contemporary UK data, we found that father absence had a delaying effect on male puberty (voice-breaking), although the effect was only apparent when father absence occurred during later childhood (we were not able to test mother absence) [92]. Although the hypotheses outlined above predict that early life stress and low parental investment should be associated with earlier puberty [1], [5]–[7], it is possible that psychological stress in general slows down male pubertal development [93]. If this is the case, then we would expect to find delayed puberty for men growing up in any of the four non-intact family situations, unless losing one parent is more stressful than losing another. It is clear that further research needs to examine how environmental factors during childhood affect male pubertal outcomes before we can gain a clearer understanding of these patterns.

### Puberty as a potential mediator

We controlled for age at puberty in all the other models as it has been shown that age at puberty is associated with age at first sex [83], marriage and total fertility [84], and risk-taking behaviour [67]. If family structure influences age at puberty (as other studies have suggested), it may be that age at puberty is a potential mediating factor which links family structure to later reproductive outcomes. It has previously been shown to be a mediator between early hardship (low maternal investment) and sexual risk-taking behaviour, although not non-sexual risk-taking behaviour [66]. Removing puberty from the models does result in stronger relationships between family structure and our reproductive and risk-taking outcomes in some cases, although in many it makes no difference. We also find that at least some correlations between family structure and life history outcomes are still significant even when controlling for puberty, suggesting that puberty does not entirely mediate the relationship between family structure and faster life history. We need to interpret this with caution however as the sample sizes increase quite dramatically when age at puberty is removed from the models. The full results for all models without age at puberty as a covariate are shown in tables S2a (for women) and S2b (for men).

### Strengths and limitations

The Kinsey data are not nationally representative [75] but provide a rare opportunity to investigate the relationship between family structure and life history strategy in a historical context in which factors like nutrition may have played more important roles than in contemporary populations (where most of the literature on this topic come from). Furthermore, the data provide excellent detail about each respondent's living arrangements throughout childhood as well as a variety of outcome variables pertaining to life history strategy in a large dataset. This allowed us the unique opportunity to examine an array of outcomes within the same population, which most other studies are not able to do. This allows for direct and meaningful comparisons between models unlike when such outcomes are compared between different studies.

We have provided here an analysis of how family structure influences outcomes in early adulthood, but there are a couple of additional factors which may influence these outcomes which we are not able to account for. The circumstances that lead to a child being in a particular family structure are an important factor that might influence their life history strategy: the effects of divorce and death may well have different consequences for children [94]. In exploratory analysis we considered grouping the data in such a way as to distinguish between children who had lost parents through divorce and death; however we would then have had double the number of family categories (e.g. single mother-death, single mother-divorce, etc), and would have had too few children represented in each category for sufficiently powerful analyses. Further complications arise since some children experienced both the divorce and death of parents, although for both sexes, parental death (∼9%) was slightly more common than divorce (∼7%). Furthermore, such analysis would become too complicated to draw meaningful conclusions from. Another issue is that of selection biases; there may well be something systematically different about, for example, women or men who remain single or remarry after widowhood or divorce. However, there are likely to be many different factors that lead to remaining single or remarried and we have no particular reason to think that those reasons would bias our results in only one direction.

## Conclusion

The current study uses a large, diverse, and methodologically rich dataset to provide one of the more complete pictures to date regarding relationships between childhood family structure and life history outcomes. We proposed that children from families other than those with both natural parents (i.e. ‘intact’ families) would receive lower levels of parental investment. Our analyses support the claim that presence of both natural parents resulted in slower life history progressions and fewer risk-taking behaviours. We extended this to argue that mother-present family types would confer higher levels of parental investment compared with father-present families due to a mother's genetic certainty and the typically higher investment of mothers compared to fathers in our species. However, our findings suggest the opposite – that father-absent (i.e. mother-based) families exerted the strongest influence on accelerating life history events in both male and female children. This demonstrates that family make-up, and in particular father absence, is likely to be an independent factor driving a faster life history, and is not merely a proxy for a low parental investment environment in general. If this were the case, then we would not have found much variation in our non-intact family categories and their associations with faster succession to these life events. Similarly, if familial disruption is a cue to mortality rates we should not have found any differences in the types of family arrangements, but we do. Our findings suggest that either there is something specific about father absence that children respond to, or perhaps that other allocarers attenuate the loss of the mother more quickly than the loss of the father. However, we are unable to distinguish between these two possibilities in a correlational study.

These findings allow us to draw two main conclusions. First, we show that different types of family construction have a unique impact on children's subsequent life history trajectories, unlike the predictions of our simple parental investment model. Secondly, we show that in this population, father absence is more important than mother absence in predicting life events. However, this is not true for age at puberty, which contradicts the predictions of most theories explaining why early life conditions should influence reproductive outcomes, at least in high-income settings. This seemingly disparate finding may be the consequence of context-dependent plasticity, signifying the importance of conducting future research to examine such associations in contexts other than high-income, low-fertility ones.

## Supporting Information

Table S1 **All results for all models for (a) women and (b) men.**
(DOCX)

Table S2 **All results for all models, excluding age at puberty covariate, for (a) women and (b) men.**
(DOCX)
