# Supplementary material for: A Not-So-Grim Tale: How Childhood Family Structure Influences Reproductive and Risk-Taking Outcomes in a Historical U.S. Population
Source: PLoS One. 2014 Mar 5;9(3):e89539. doi: 10.1371/journal.pone.0089539 (PMC3943735; doi:10.1371/journal.pone.0089539)
Supplement: Table S1 — All results for all models for (a) women and (b) men. (DOCX) [file pone.0089539.s001.docx]

**ELECTRONIC SUPPLEMENTARY MATERIAL**

| Table S1a: all results for all models for women | | |  |  |  |  |  |
| --- | --- | --- | --- | --- | --- | --- | --- |
| **AGE AT PUBERTY¹** | **Coef.** | **S.E.** | **t** | **P>t** | **95% C.I.** | | **n=4624** |
| *ref: intact family* |  |  |  |  |  |  |  |
| SINGLE DAD | 0.06 | 0.152 | 0.380 | 0.705 | -0.24 | 0.36 |  |
| SINGLE MUM | -0.03 | 0.072 | -0.450 | 0.650 | -0.17 | 0.11 |  |
| DAD + STEPMUM | -0.04 | 0.132 | -0.330 | 0.740 | -0.30 | 0.22 |  |
| MUM + STEPDAD | -0.07 | 0.122 | -0.550 | 0.584 | -0.31 | 0.17 |  |
| FOSTER NON-RELATIVES | 0.03 | 0.232 | 0.150 | 0.884 | -0.42 | 0.49 |  |
| FOSTER RELATIVES | 0.25 | 0.151 | 1.650 | 0.098 | -0.05 | 0.54 |  |
| INSTITUTION | 0.40 | 0.233 | 1.730 | 0.083 | -0.05 | 0.86 |  |
| year of birth | -0.02 | 0.002 | -8.520 | 0.000 | -0.02 | -0.01 |  |
| white | 0.25 | 0.063 | 3.980 | 0.000 | 0.13 | 0.37 |  |
| socioeconomic status | -0.05 | 0.014 | -3.280 | 0.001 | -0.07 | -0.02 |  |
| family size | 0.11 | 0.012 | 9.000 | 0.000 | 0.08 | 0.13 |  |
| birth order | 0.07 | 0.030 | 2.330 | 0.020 | 0.01 | 0.13 |  |
| birth order² | -0.01 | 0.003 | -4.400 | 0.000 | -0.02 | -0.01 |  |
| intercept | 44.54 | 3.839 | 11.600 | 0.000 | 37.01 | 52.06 |  |
|  |  |  |  |  |  |  |  |
| **AGE AT 1ST PETTING¹** | **Coef.** | **S.E.** | **t** | **P>t** | **95% C.I.** | | **n=4413** |
| *ref: intact family* |  |  |  |  |  |  |  |
| SINGLE DAD | -0.22 | 0.294 | -0.750 | 0.453 | -0.80 | 0.36 |  |
| SINGLE MUM | -0.24 | 0.139 | -1.750 | 0.080 | -0.51 | 0.03 |  |
| DAD + STEPMUM | -0.16 | 0.264 | -0.610 | 0.539 | -0.68 | 0.36 |  |
| MUM + STEPDAD | -0.65 | 0.236 | -2.770 | 0.006 | -1.12 | -0.19 |  |
| FOSTER NON-RELATIVES | -0.75 | 0.452 | -1.650 | 0.099 | -1.63 | 0.14 |  |
| FOSTER RELATIVES | -0.48 | 0.290 | -1.640 | 0.102 | -1.04 | 0.09 |  |
| INSTITUTION | 0.28 | 0.463 | 0.600 | 0.552 | -0.63 | 1.18 |  |
| year of birth | -0.06 | 0.004 | -15.200 | 0.000 | -0.07 | -0.05 |  |
| white | 0.95 | 0.123 | 7.760 | 0.000 | 0.71 | 1.19 |  |
| socioeconomic status | 0.15 | 0.027 | 5.430 | 0.000 | 0.09 | 0.20 |  |
| age at puberty | 0.33 | 0.029 | 11.450 | 0.000 | 0.27 | 0.39 |  |
| family size | 0.02 | 0.024 | 0.720 | 0.471 | -0.03 | 0.06 |  |
| birth order | -0.09 | 0.058 | -1.500 | 0.132 | -0.20 | 0.03 |  |
| birth order² | 0.00 | 0.006 | -0.040 | 0.969 | -0.01 | 0.01 |  |
| intercept | 125.21 | 7.596 | 16.480 | 0.000 | 110.32 | 140.10 |  |

|  |  | |  |  |  |  |  |  |
| --- | --- | --- | --- | --- | --- | --- | --- | --- |
| **PROGRESSION TO 1ST SEX²** | **O.R.** | | **S.E.** | **z** | **P>z** | **95% C.I.** | | **n=4622** |
| SINGLE DAD | 1.87 | | 0.274 | 4.280 | 0.000 | 1.40 | 2.49 |  |
| SINGLE MUM | 6.13 | | 1.803 | 6.170 | 0.000 | 3.45 | 10.91 |  |
| DAD + STEPMUM | 1.31 | | 0.172 | 2.020 | 0.043 | 1.01 | 1.69 |  |
| MUM + STEPDAD | 6.27 | | 1.678 | 6.860 | 0.000 | 3.71 | 10.59 |  |
| foster | 3.02 | | 0.358 | 9.310 | 0.000 | 2.39 | 3.81 |  |
| INSTITUTION | 2.31 | | 0.507 | 3.830 | 0.000 | 1.51 | 3.55 |  |
| time | 2.77 | | 0.090 | 31.500 | 0.000 | 2.60 | 2.95 |  |
| time² | 0.98 | | 0.001 | -26.300 | 0.000 | 0.98 | 0.98 |  |
| SINGLE MUM*TIME | 0.92 | | 0.015 | -5.130 | 0.000 | 0.89 | 0.95 |  |
| MUM+STEPDAD*TIME² | 1.00 | | 0.001 | -3.540 | 0.000 | 1.00 | 1.00 |  |
| year of birth | 0.82 | | 0.063 | -2.590 | 0.010 | 0.70 | 0.95 |  |
| age | 0.98 | | 0.007 | -3.660 | 0.000 | 0.96 | 0.99 |  |
| white | 0.41 | | 0.025 | -14.600 | 0.000 | 0.36 | 0.46 |  |
| socioeconomic status | 0.82 | | 0.012 | -13.700 | 0.000 | 0.80 | 0.85 |  |
| age at puberty | 0.98 | | 0.015 | -1.180 | 0.238 | 0.95 | 1.01 |  |
| family size | 1.08 | | 0.013 | 6.390 | 0.000 | 1.05 | 1.10 |  |
| birth order | 0.94 | | 0.028 | -2.100 | 0.036 | 0.88 | 1.00 |  |
| birth order² | 1.01 | | 0.003 | 2.020 | 0.043 | 1.00 | 1.01 |  |
| intercept | 0.00 | | 0.000 | -27.030 | 0.000 | 0.00 | 0.00 |  |
|  |  | |  |  |  |  |  |  |
| **ANY PREMARITAL SEX³** | **O.R.** | **S.E.** | | **z** | **P>z** | **95% C.I.** | | **n=2992** |
| *ref: intact family* |  |  | |  |  |  |  |  |
| SINGLE DAD | 1.41 | 0.428 | | 1.140 | 0.256 | 0.78 | 2.56 |  |
| SINGLE MUM | 1.44 | 0.227 | | 2.330 | 0.020 | 1.06 | 1.96 |  |
| DAD + STEPMUM | 0.80 | 0.224 | | -0.780 | 0.436 | 0.47 | 1.39 |  |
| MUM + STEPDAD | 1.05 | 0.255 | | 0.200 | 0.838 | 0.65 | 1.69 |  |
| FOSTER NON-RELATIVES | 2.21 | 1.238 | | 1.420 | 0.156 | 0.74 | 6.62 |  |
| FOSTER RELATIVES | 0.86 | 0.247 | | -0.540 | 0.589 | 0.49 | 1.51 |  |
| INSTITUTION | 10.41 | 10.741 | | 2.270 | 0.023 | 1.38 | 78.64 |  |
| year of birth | 1.06 | 0.004 | | 13.250 | 0.000 | 1.05 | 1.06 |  |
| white | 0.28 | 0.044 | | -8.140 | 0.000 | 0.21 | 0.38 |  |
| socioeconomic status | 1.01 | 0.028 | | 0.400 | 0.686 | 0.96 | 1.07 |  |
| age at puberty | 0.95 | 0.029 | | -1.700 | 0.089 | 0.89 | 1.01 |  |
| family size | 1.02 | 0.025 | | 0.840 | 0.401 | 0.97 | 1.07 |  |
| birth order | 0.82 | 0.057 | | -2.800 | 0.005 | 0.72 | 0.94 |  |
| birth order² | 1.02 | 0.008 | | 2.660 | 0.008 | 1.01 | 1.04 |  |
| intercept | 0.00 | 0.000 | | -12.860 | 0.000 | 0.00 | 0.00 |  |

|  |  |  |  |  |  |  |  |
| --- | --- | --- | --- | --- | --- | --- | --- |
| **NUMBER OF SEX PARTNERS^4^** | **R.R.R.** | **S.E.** | **z** | **P>z** | **95% C.I.** | | **n=2992** |
| *base outcome: none* |  |  |  |  |  |  |  |
| **1 to 5 partners** |  |  |  |  |  |  |  |
| *ref: intact family* |  |  |  |  |  |  |  |
| SINGLE DAD | 1.37 | 0.429 | 1.010 | 0.311 | 0.74 | 2.53 |  |
| SINGLE MUM | 1.40 | 0.227 | 2.090 | 0.037 | 1.02 | 1.93 |  |
| DAD + STEPMUM | 0.77 | 0.224 | -0.890 | 0.371 | 0.44 | 1.36 |  |
| MUM + STEPDAD | 1.01 | 0.256 | 0.060 | 0.953 | 0.62 | 1.66 |  |
| FOSTER NON-RELATIVES | 1.44 | 0.859 | 0.600 | 0.546 | 0.44 | 4.64 |  |
| FOSTER RELATIVES | 0.89 | 0.261 | -0.410 | 0.679 | 0.50 | 1.58 |  |
| INSTITUTION | 8.91 | 9.284 | 2.100 | 0.036 | 1.15 | 68.71 |  |
| year of birth | 1.05 | 0.004 | 12.040 | 0.000 | 1.04 | 1.06 |  |
| white | 0.31 | 0.049 | -7.460 | 0.000 | 0.22 | 0.42 |  |
| socioeconomic status | 1.04 | 0.030 | 1.230 | 0.217 | 0.98 | 1.10 |  |
| age at puberty | 0.95 | 0.030 | -1.790 | 0.074 | 0.89 | 1.01 |  |
| family size | 1.01 | 0.025 | 0.310 | 0.753 | 0.96 | 1.06 |  |
| birth order | 0.84 | 0.060 | -2.420 | 0.016 | 0.73 | 0.97 |  |
| birth order² | 1.02 | 0.008 | 2.510 | 0.012 | 1.00 | 1.03 |  |
| intercept | 0.00 | 0.000 | -11.700 | 0.000 | 0.00 | 0.00 |  |
| **6+ partners** |  |  |  |  |  |  |  |
| *ref: intact family* |  |  |  |  |  |  |  |
| SINGLE DAD | 1.61 | 0.702 | 1.100 | 0.273 | 0.69 | 3.78 |  |
| SINGLE MUM | 1.62 | 0.345 | 2.250 | 0.024 | 1.06 | 2.46 |  |
| DAD + STEPMUM | 0.94 | 0.360 | -0.160 | 0.875 | 0.44 | 1.99 |  |
| MUM + STEPDAD | 1.20 | 0.394 | 0.540 | 0.588 | 0.63 | 2.28 |  |
| FOSTER NON-RELATIVES | 5.37 | 3.278 | 2.760 | 0.006 | 1.62 | 17.76 |  |
| FOSTER RELATIVES | 0.73 | 0.324 | -0.710 | 0.478 | 0.30 | 1.74 |  |
| INSTITUTION | 15.73 | 16.969 | 2.550 | 0.011 | 1.90 | 130.29 |  |
| year of birth | 1.07 | 0.007 | 10.230 | 0.000 | 1.06 | 1.09 |  |
| white | 0.22 | 0.041 | -8.030 | 0.000 | 0.15 | 0.32 |  |
| socioeconomic status | 0.91 | 0.037 | -2.290 | 0.022 | 0.84 | 0.99 |  |
| age at puberty | 0.97 | 0.044 | -0.710 | 0.476 | 0.89 | 1.06 |  |
| family size | 1.07 | 0.037 | 1.950 | 0.051 | 1.00 | 1.14 |  |
| birth order | 0.75 | 0.071 | -3.040 | 0.002 | 0.62 | 0.90 |  |
| birth order² | 1.02 | 0.010 | 2.550 | 0.011 | 1.01 | 1.04 |  |
| intercept | 0.00 | 0.000 | -10.070 | 0.000 | 0.00 | 0.00 |  |

|  |  |  |  |  |  |  |  |
| --- | --- | --- | --- | --- | --- | --- | --- |
| **PROGRESSION TO MARRIAGE²** | **O.R.** | **S.E.** | **z** | **P>z** | **95% C.I.** | | **n=4612** |
| *ref: intact family* |  |  |  |  |  |  |  |
| SINGLE DAD | 1.46 | 0.238 | 2.330 | 0.020 | 1.06 | 2.01 |  |
| SINGLE MUM | 1.10 | 0.090 | 1.160 | 0.245 | 0.94 | 1.29 |  |
| DAD + STEPMUM | 1.30 | 0.194 | 1.730 | 0.083 | 0.97 | 1.74 |  |
| MUM + STEPDAD | 1.69 | 0.220 | 4.030 | 0.000 | 1.31 | 2.18 |  |
| FOSTER NON-RELATIVES | 2.53 | 0.649 | 3.610 | 0.000 | 1.53 | 4.18 |  |
| FOSTER RELATIVES | 2.03 | 0.307 | 4.700 | 0.000 | 1.51 | 2.73 |  |
| INSTITUTION | 0.88 | 0.268 | -0.420 | 0.677 | 0.49 | 1.60 |  |
| time | 3.38 | 0.179 | 23.000 | 0.000 | 3.05 | 3.75 |  |
| time² | 0.98 | 0.001 | -20.000 | 0.000 | 0.97 | 0.98 |  |
| age | 0.98 | 0.008 | -2.030 | 0.043 | 0.97 | 1.00 |  |
| year of birth | 0.75 | 0.066 | -3.260 | 0.001 | 0.63 | 0.89 |  |
| white | 0.83 | 0.058 | -2.630 | 0.009 | 0.72 | 0.95 |  |
| socioeconomic status | 0.83 | 0.013 | -11.430 | 0.000 | 0.81 | 0.86 |  |
| age at puberty | 0.99 | 0.017 | -0.440 | 0.662 | 0.96 | 1.03 |  |
| family size | 1.05 | 0.014 | 3.680 | 0.000 | 1.02 | 1.08 |  |
| birth order | 0.97 | 0.034 | -0.840 | 0.399 | 0.91 | 1.04 |  |
| birth order² | 1.00 | 0.003 | 0.360 | 0.719 | 0.99 | 1.01 |  |
| intercept | 0.00 | 0.000 | -24.110 | 0.000 | 0.00 | 0.00 |  |
|  |  |  |  |  |  |  |  |
| **MORE THAN ONE MARRIAGE³** | **O.R.** | **S.E.** | **z** | **P>z** | **95% C.I.** | | **n=2193** |
| *ref: intact family* |  |  |  |  |  |  |  |
| SINGLE DAD | 1.01 | 0.391 | 0.040 | 0.971 | 0.48 | 2.16 |  |
| SINGLE MUM | 1.18 | 0.233 | 0.860 | 0.390 | 0.81 | 1.74 |  |
| DAD + STEPMUM | 1.22 | 0.461 | 0.530 | 0.596 | 0.58 | 2.56 |  |
| MUM + STEPDAD | 1.52 | 0.453 | 1.410 | 0.159 | 0.85 | 2.73 |  |
| FOSTER NON-RELATIVES | 2.21 | 1.353 | 1.300 | 0.193 | 0.67 | 7.34 |  |
| FOSTER RELATIVES | 1.74 | 0.578 | 1.670 | 0.095 | 0.91 | 3.34 |  |
| INSTITUTION | 0.91 | 0.727 | -0.120 | 0.905 | 0.19 | 4.36 |  |
| white | 1.03 | 0.179 | 0.180 | 0.854 | 0.74 | 1.45 |  |
| year of birth | 0.54 | 0.031 | -10.540 | 0.000 | 0.49 | 0.61 |  |
| socioeconomic status | 0.86 | 0.032 | -4.030 | 0.000 | 0.80 | 0.93 |  |
| age at puberty | 0.92 | 0.039 | -2.070 | 0.038 | 0.84 | 1.00 |  |
| family size | 1.01 | 0.032 | 0.200 | 0.839 | 0.95 | 1.07 |  |
| birth order | 0.94 | 0.076 | -0.790 | 0.430 | 0.80 | 1.10 |  |
| birth order² | 1.01 | 0.008 | 1.680 | 0.093 | 1.00 | 1.03 |  |
| intercept | 0.86 | 0.477 | -0.280 | 0.781 | 0.29 | 2.55 |  |

|  |  |  |  |  |  |  |  |
| --- | --- | --- | --- | --- | --- | --- | --- |
| **AGE AT 1ST BIRTH¹** | **Coef.** | **S.E.** | **t** | **P>t** | **95% CI** | | **n=699** |
| *ref: intact family* |  |  |  |  |  |  |  |
| SINGLE DAD | -34.92 | 16.276 | -2.150 | 0.032 | -66.88 | -2.97 |  |
| SINGLE MUM | -9.81 | 8.079 | -1.210 | 0.225 | -25.67 | 6.05 |  |
| DAD + STEPMUM | 1.93 | 15.412 | 0.130 | 0.900 | -28.33 | 32.19 |  |
| MUM + STEPDAD | -49.97 | 15.656 | -3.190 | 0.001 | -80.71 | -19.23 |  |
| FOSTER NON-RELATIVES | -95.83 | 58.119 | -1.650 | 0.100 | -209.94 | 18.29 |  |
| FOSTER RELATIVES | -16.05 | 14.839 | -1.080 | 0.280 | -45.19 | 13.09 |  |
| INSTITUTION (omitted) |  |  |  |  |  |  |  |
| year of birth | -0.02 | 0.245 | -0.100 | 0.921 | -0.51 | 0.46 |  |
| white | 23.45 | 7.946 | 2.950 | 0.003 | 7.85 | 39.05 |  |
| socioeconomic status | 7.48 | 1.420 | 5.270 | 0.000 | 4.69 | 10.27 |  |
| age at puberty | 2.53 | 1.576 | 1.600 | 0.109 | -0.57 | 5.62 |  |
| family size | -3.63 | 1.114 | -3.260 | 0.001 | -5.81 | -1.44 |  |
| birth order | -2.11 | 3.407 | -0.620 | 0.536 | -8.80 | 4.58 |  |
| birth order² | 0.06 | 0.363 | 0.160 | 0.876 | -0.66 | 0.77 |  |
| intercept | 262.60 | 469.225 | 0.560 | 0.576 | -658.69 | 1183.89 |  |
|  |  |  |  |  |  |  |  |
| **ANY EXTRAMARITAL SEX³** | **O.R.** | **S.E.** | **z** | **P>z** | **95% C.I.** | |  |
| *ref: intact family* |  |  |  |  |  |  |  |
| SINGLE DAD | 0.93 | 0.319 | -0.200 | 0.841 | 0.48 | 1.82 |  |
| SINGLE MUM | 1.01 | 0.173 | 0.070 | 0.944 | 0.72 | 1.42 |  |
| DAD + STEPMUM | 0.58 | 0.208 | -1.520 | 0.128 | 0.29 | 1.17 |  |
| MUM + STEPDAD | 1.08 | 0.281 | 0.310 | 0.754 | 0.65 | 1.80 |  |
| FOSTER NON-RELATIVES | 1.73 | 0.849 | 1.120 | 0.264 | 0.66 | 4.53 |  |
| FOSTER RELATIVES | 1.67 | 0.468 | 1.840 | 0.066 | 0.97 | 2.90 |  |
| INSTITUTION | 0.87 | 0.581 | -0.210 | 0.837 | 0.24 | 3.22 |  |
| year of birth | 1.01 | 0.052 | 0.110 | 0.913 | 0.91 | 1.11 |  |
| white | 0.61 | 0.087 | -3.430 | 0.001 | 0.47 | 0.81 |  |
| socioeconomic status | 1.04 | 0.033 | 1.100 | 0.270 | 0.97 | 1.10 |  |
| age at puberty | 1.01 | 0.036 | 0.230 | 0.819 | 0.94 | 1.08 |  |
| family size | 0.96 | 0.028 | -1.370 | 0.171 | 0.91 | 1.02 |  |
| birth order | 0.94 | 0.068 | -0.810 | 0.419 | 0.82 | 1.09 |  |
| birth order² | 1.01 | 0.007 | 0.950 | 0.341 | 0.99 | 1.02 |  |
| intercept | 0.51 | 0.241 | -1.430 | 0.154 | 0.20 | 1.29 |  |

|  |  |  |  |  |  |  |  |
| --- | --- | --- | --- | --- | --- | --- | --- |
| **GAMBLING^4^** | **R.R.R.** | **S.E.** | **z** | **P>z** | **95% C.I.** | | **n=4464** |
| **a little** |  |  |  |  |  |  |  |
| *ref: intact family* |  |  |  |  |  |  |  |
| SINGLE DAD | 0.85 | 0.276 | -0.510 | 0.610 | 0.45 | 1.60 |  |
| SINGLE MUM | 1.21 | 0.171 | 1.370 | 0.171 | 0.92 | 1.60 |  |
| DAD + STEPMUM | 0.87 | 0.245 | -0.510 | 0.613 | 0.50 | 1.51 |  |
| MUM + STEPDAD | 1.11 | 0.283 | 0.410 | 0.681 | 0.67 | 1.83 |  |
| FOSTER NON-RELATIVES | 0.97 | 0.488 | -0.060 | 0.955 | 0.36 | 2.60 |  |
| FOSTER RELATIVES | 0.54 | 0.224 | -1.480 | 0.138 | 0.24 | 1.22 |  |
| INSTITUTION | 2.17 | 0.948 | 1.770 | 0.076 | 0.92 | 5.11 |  |
| year of birth | 0.88 | 0.038 | -2.900 | 0.004 | 0.81 | 0.96 |  |
| white | 0.80 | 0.106 | -1.710 | 0.087 | 0.61 | 1.03 |  |
| socioeconomic status | 1.18 | 0.034 | 5.800 | 0.000 | 1.12 | 1.25 |  |
| age at puberty | 0.95 | 0.029 | -1.560 | 0.119 | 0.90 | 1.01 |  |
| family size | 0.94 | 0.025 | -2.270 | 0.023 | 0.89 | 0.99 |  |
| birth order | 1.06 | 0.069 | 0.920 | 0.360 | 0.93 | 1.21 |  |
| birth order² | 1.00 | 0.007 | -0.130 | 0.894 | 0.99 | 1.01 |  |
| intercept | 0.28 | 0.115 | -3.110 | 0.002 | 0.13 | 0.62 |  |
| **more than a little** |  |  |  |  |  |  |  |
| *ref: intact family* |  |  |  |  |  |  |  |
| SINGLE DAD | 1.42 | 0.596 | 0.840 | 0.402 | 0.62 | 3.23 |  |
| SINGLE MUM | 1.27 | 0.266 | 1.150 | 0.251 | 0.84 | 1.91 |  |
| DAD + STEPMUM | 0.53 | 0.277 | -1.220 | 0.223 | 0.19 | 1.48 |  |
| MUM + STEPDAD | 2.22 | 0.646 | 2.730 | 0.006 | 1.25 | 3.93 |  |
| FOSTER NON-RELATIVES | 1.58 | 1.004 | 0.720 | 0.470 | 0.46 | 5.49 |  |
| FOSTER RELATIVES | 4.70 | 1.332 | 5.470 | 0.000 | 2.70 | 8.19 |  |
| INSTITUTION | 3.36 | 1.767 | 2.310 | 0.021 | 1.20 | 9.42 |  |
| year of birth | 0.88 | 0.058 | -1.880 | 0.060 | 0.78 | 1.01 |  |
| white | 0.36 | 0.056 | -6.510 | 0.000 | 0.27 | 0.49 |  |
| socioeconomic status | 0.93 | 0.040 | -1.760 | 0.079 | 0.85 | 1.01 |  |
| age at puberty | 0.96 | 0.046 | -0.780 | 0.433 | 0.88 | 1.06 |  |
| family size | 0.97 | 0.038 | -0.820 | 0.414 | 0.90 | 1.05 |  |
| birth order | 1.06 | 0.091 | 0.690 | 0.488 | 0.90 | 1.26 |  |
| birth order² | 1.00 | 0.007 | 0.650 | 0.517 | 0.99 | 1.02 |  |
| intercept | 0.37 | 0.233 | -1.580 | 0.114 | 0.11 | 1.27 |  |

|  |  |  |  |  |  |  |  |
| --- | --- | --- | --- | --- | --- | --- | --- |
| **USED ILLEGAL DRUGS³** | **O.R.** | **S.E.** | **z** | **P>z** | **95% C.I.** | | **n=4422** |
| *ref: intact family* |  |  |  |  |  |  |  |
| SINGLE DAD | 1.35 | 0.743 | 0.550 | 0.580 | 0.46 | 3.97 |  |
| SINGLE MUM | 1.62 | 0.385 | 2.030 | 0.043 | 1.02 | 2.58 |  |
| DAD + STEPMUM | 1.67 | 0.710 | 1.200 | 0.231 | 0.72 | 3.84 |  |
| MUM + STEPDAD | 2.69 | 0.893 | 2.980 | 0.003 | 1.40 | 5.16 |  |
| FOSTER NON-RELATIVES | 4.67 | 2.487 | 2.890 | 0.004 | 1.64 | 13.26 |  |
| FOSTER RELATIVES | 3.88 | 1.351 | 3.890 | 0.000 | 1.96 | 7.68 |  |
| INSTITUTION | 2.19 | 1.400 | 1.230 | 0.220 | 0.63 | 7.66 |  |
| year of birth | 1.06 | 0.096 | 0.620 | 0.538 | 0.89 | 1.26 |  |
| white | 0.30 | 0.055 | -6.520 | 0.000 | 0.21 | 0.43 |  |
| socioeconomic status | 0.74 | 0.040 | -5.580 | 0.000 | 0.66 | 0.82 |  |
| age at puberty | 1.03 | 0.063 | 0.550 | 0.582 | 0.92 | 1.17 |  |
| family size | 0.87 | 0.049 | -2.450 | 0.014 | 0.78 | 0.97 |  |
| birth order | 1.05 | 0.115 | 0.420 | 0.677 | 0.84 | 1.30 |  |
| birth order² | 1.01 | 0.009 | 1.540 | 0.123 | 1.00 | 1.03 |  |
| intercept | 0.23 | 0.178 | -1.890 | 0.059 | 0.05 | 1.06 |  |

¹ linear regression analyses, ² discrete-time event-history analyses, ³ binary logistic regression analyses, ^4^ multinomial logistic analyses

Coef. = beta coefficient; O.R. = odds ratio; R.R.R = relative risk ratio, S.E. = standard error; C.I. = confidence interval

| Table S1b: All results for all models for men | | | |  |  |  |  |  |
| --- | --- | --- | --- | --- | --- | --- | --- | --- |
| **AGE AT PUBERTY¹** | **Coef.** | | **S.E.** | **t** | **P>t** | **95% C.I.** | | **n=4749** |
| *ref: intact family* |  | |  |  |  |  |  |  |
| SINGLE DAD | 0.31 | | 0.142 | 2.200 | 0.028 | 0.03 | 0.59 |  |
| SINGLE MUM | 0.12 | | 0.075 | 1.620 | 0.104 | -0.03 | 0.27 |  |
| DAD + STEPMUM | 0.35 | | 0.152 | 2.330 | 0.020 | 0.06 | 0.65 |  |
| MUM + STEPDAD | 0.18 | | 0.110 | 1.600 | 0.109 | -0.04 | 0.39 |  |
| FOSTER NON-RELATIVES | -0.13 | | 0.213 | -0.600 | 0.547 | -0.55 | 0.29 |  |
| FOSTER RELATIVES | 0.28 | | 0.140 | 1.970 | 0.049 | 0.00 | 0.55 |  |
| INSTITUTION | 0.50 | | 0.185 | 2.680 | 0.007 | 0.13 | 0.86 |  |
| year of birth | -0.02 | | 0.002 | -9.070 | 0.000 | -0.02 | -0.01 |  |
| white | -0.60 | | 0.061 | -9.860 | 0.000 | -0.72 | -0.48 |  |
| socioeconomic status | -0.06 | | 0.014 | -4.620 | 0.000 | -0.09 | -0.04 |  |
| family size | 0.05 | | 0.012 | 4.460 | 0.000 | 0.03 | 0.07 |  |
| birth order | 0.00 | | 0.031 | -0.030 | 0.978 | -0.06 | 0.06 |  |
| birth order² | 0.00 | | 0.003 | -0.650 | 0.517 | -0.01 | 0.00 |  |
| intercept | 47.98 | | 3.747 | 12.800 | 0.000 | 40.64 | 55.33 |  |
|  |  | |  |  |  |  |  |  |
| **AGE AT 1ST PETTING¹** | | **Coef.** | **S.E.** | **t** | **P>t** | **95% C.I.** | | **n=4582** |
| *ref: intact family* | |  |  |  |  |  |  |  |
| SINGLE DAD | | -0.20 | 0.297 | -0.690 | 0.491 | -0.79 | 0.38 |  |
| SINGLE MUM | | -0.16 | 0.159 | -0.980 | 0.328 | -0.47 | 0.16 |  |
| DAD + STEPMUM | | -0.24 | 0.318 | -0.760 | 0.447 | -0.87 | 0.38 |  |
| MUM + STEPDAD | | -0.29 | 0.232 | -1.230 | 0.220 | -0.74 | 0.17 |  |
| FOSTER NON-RELATIVES | | 0.00 | 0.457 | 0.000 | 0.997 | -0.90 | 0.89 |  |
| FOSTER RELATIVES | | -0.42 | 0.298 | -1.410 | 0.157 | -1.01 | 0.16 |  |
| INSTITUTION | | 0.38 | 0.387 | 0.990 | 0.323 | -0.38 | 1.14 |  |
| year of birth | | -0.05 | 0.004 | -13.130 | 0.000 | -0.06 | -0.05 |  |
| white | | 1.50 | 0.130 | 11.560 | 0.000 | 1.25 | 1.76 |  |
| socioeconomic status | | 0.03 | 0.029 | 0.960 | 0.339 | -0.03 | 0.08 |  |
| age at puberty | | 0.54 | 0.031 | 17.570 | 0.000 | 0.48 | 0.60 |  |
| family size | | -0.05 | 0.024 | -2.030 | 0.042 | -0.10 | 0.00 |  |
| birth order | | -0.04 | 0.067 | -0.550 | 0.582 | -0.17 | 0.09 |  |
| birth order² | | 0.00 | 0.007 | 0.560 | 0.578 | -0.01 | 0.02 |  |
| intercept | | 111.78 | 8.054 | 13.880 | 0.000 | 95.99 | 127.56 |  |

|  |  |  |  |  |  |  |  |
| --- | --- | --- | --- | --- | --- | --- | --- |
| **PROGRESSION TO 1ST SEX²** | **O.R.** | **S.E.** | **z** | **P>z** | **95% C.I.** | | **n=4744** |
| SINGLE DAD | 1.29 | 0.166 | 1.970 | 0.048 | 1.00 | 1.66 |  |
| SINGLE MUM | 0.19 | 0.222 | -1.430 | 0.152 | 0.02 | 1.83 |  |
| DAD + STEPMUM | 1.08 | 0.152 | 0.540 | 0.593 | 0.82 | 1.42 |  |
| MUM + STEPDAD | 1.28 | 0.128 | 2.480 | 0.013 | 1.05 | 1.56 |  |
| FOSTER NON-REL | 1.39 | 0.267 | 1.740 | 0.082 | 0.96 | 2.03 |  |
| FOSTER REL | 1.04 | 0.138 | 0.320 | 0.750 | 0.80 | 1.35 |  |
| INSTITUTION | 1.37 | 0.235 | 1.820 | 0.069 | 0.98 | 1.92 |  |
| time | 2.53 | 0.079 | 29.730 | 0.000 | 2.38 | 2.69 |  |
| time² | 0.98 | 0.001 | -25.100 | 0.000 | 0.98 | 0.98 |  |
| SINGLE MUM*TIME | 1.33 | 0.179 | 2.140 | 0.032 | 1.02 | 1.73 |  |
| SINGLE MUM*TIME² | 0.99 | 0.004 | -2.680 | 0.007 | 0.98 | 1.00 |  |
| year of birth | 1.05 | 0.048 | 1.110 | 0.265 | 0.96 | 1.15 |  |
| age | 1.00 | 0.004 | 0.680 | 0.494 | 1.00 | 1.01 |  |
| white | 0.34 | 0.019 | -18.890 | 0.000 | 0.30 | 0.38 |  |
| socioeconomic status | 0.91 | 0.012 | -7.200 | 0.000 | 0.89 | 0.94 |  |
| age at puberty | 0.95 | 0.012 | -4.350 | 0.000 | 0.92 | 0.97 |  |
| family size | 1.07 | 0.012 | 6.400 | 0.000 | 1.05 | 1.10 |  |
| birth order | 1.04 | 0.029 | 1.520 | 0.129 | 0.99 | 1.10 |  |
| birth order² | 0.99 | 0.003 | -2.180 | 0.030 | 0.99 | 1.00 |  |
| intercept | 0.00 | 0.000 | -26.600 | 0.000 | 0.00 | 0.00 |  |
|  |  |  |  |  |  |  |  |
| **ANY PREMARITAL SEX³** | **O.R.** | **S.E.** | **z** | **P>z** | **95% C.I.** | | **n=3654** |
| *ref: intact family* |  |  |  |  |  |  |  |
| SINGLE DAD | 1.19 | 0.465 | 0.440 | 0.662 | 0.55 | 2.56 |  |
| SINGLE MUM | 1.43 | 0.330 | 1.550 | 0.122 | 0.91 | 2.25 |  |
| DAD + STEPMUM | 1.09 | 0.459 | 0.190 | 0.847 | 0.47 | 2.48 |  |
| MUM + STEPDAD | 2.81 | 1.307 | 2.220 | 0.027 | 1.13 | 6.99 |  |
| FOSTER NON-RELATIVES | 0.63 | 0.306 | -0.950 | 0.344 | 0.25 | 1.63 |  |
| FOSTER RELATIVES | 2.38 | 1.254 | 1.650 | 0.100 | 0.85 | 6.69 |  |
| INSTITUTION | 2.18 | 1.415 | 1.200 | 0.232 | 0.61 | 7.79 |  |
| year of birth | 1.04 | 0.005 | 9.040 | 0.000 | 1.03 | 1.05 |  |
| white | 0.17 | 0.052 | -5.820 | 0.000 | 0.09 | 0.31 |  |
| socioeconomic status | 0.95 | 0.035 | -1.300 | 0.194 | 0.89 | 1.02 |  |
| age at puberty | 0.99 | 0.038 | -0.290 | 0.770 | 0.92 | 1.07 |  |
| family size | 1.14 | 0.040 | 3.640 | 0.000 | 1.06 | 1.22 |  |
| birth order | 1.00 | 0.083 | -0.020 | 0.984 | 0.85 | 1.17 |  |
| birth order² | 0.99 | 0.008 | -1.090 | 0.274 | 0.97 | 1.01 |  |
| intercept | 0.00 | 0.000 | -8.540 | 0.000 | 0.00 | 0.00 |  |

|  |  |  |  |  |  |  |  |
| --- | --- | --- | --- | --- | --- | --- | --- |
| **NUMBER OF SEX PARTNERS^4^** | **R.R.R.** | **S.E.** | **z** | **P>z** | **95% C.I.** | | **n=3654** |
| *base outcome: none* |  |  |  |  |  |  |  |
| **1 to 10 partners** |  |  |  |  |  |  |  |
| *ref: intact family* |  |  |  |  |  |  |  |
| SINGLE DAD | 0.98 | 0.399 | -0.060 | 0.952 | 0.44 | 2.18 |  |
| SINGLE MUM | 1.34 | 0.318 | 1.250 | 0.213 | 0.84 | 2.14 |  |
| DAD + STEPMUM | 1.19 | 0.514 | 0.410 | 0.679 | 0.51 | 2.77 |  |
| MUM + STEPDAD | 2.50 | 1.180 | 1.940 | 0.052 | 0.99 | 6.31 |  |
| FOSTER NON-RELATIVES | 0.52 | 0.268 | -1.260 | 0.207 | 0.19 | 1.43 |  |
| FOSTER RELATIVES | 2.00 | 1.076 | 1.290 | 0.197 | 0.70 | 5.74 |  |
| INSTITUTION | 2.17 | 1.433 | 1.170 | 0.242 | 0.59 | 7.92 |  |
| year of birth | 1.04 | 0.005 | 9.040 | 0.000 | 1.03 | 1.05 |  |
| white | 0.29 | 0.091 | -3.960 | 0.000 | 0.16 | 0.54 |  |
| socioeconomic status | 0.99 | 0.037 | -0.390 | 0.698 | 0.92 | 1.06 |  |
| age at puberty | 0.98 | 0.039 | -0.390 | 0.695 | 0.91 | 1.06 |  |
| family size | 1.12 | 0.040 | 3.130 | 0.002 | 1.04 | 1.20 |  |
| birth order | 0.96 | 0.081 | -0.480 | 0.629 | 0.81 | 1.13 |  |
| birth order² | 1.00 | 0.009 | -0.550 | 0.580 | 0.98 | 1.01 |  |
| intercept | 0.00 | 0.000 | -8.650 | 0.000 | 0.00 | 0.00 |  |
| **10+ partners** |  |  |  |  |  |  |  |
| *ref: intact family* |  |  |  |  |  |  |  |
| SINGLE DAD | 1.67 | 0.704 | 1.210 | 0.225 | 0.73 | 3.81 |  |
| SINGLE MUM | 1.61 | 0.401 | 1.920 | 0.055 | 0.99 | 2.63 |  |
| DAD + STEPMUM | 0.83 | 0.414 | -0.370 | 0.714 | 0.32 | 2.21 |  |
| MUM + STEPDAD | 3.48 | 1.674 | 2.590 | 0.010 | 1.35 | 8.93 |  |
| FOSTER NON-RELATIVES | 0.87 | 0.461 | -0.270 | 0.787 | 0.30 | 2.46 |  |
| FOSTER RELATIVES | 3.28 | 1.803 | 2.160 | 0.031 | 1.12 | 9.64 |  |
| INSTITUTION | 2.20 | 1.516 | 1.150 | 0.251 | 0.57 | 8.49 |  |
| year of birth | 1.04 | 0.006 | 7.190 | 0.000 | 1.03 | 1.05 |  |
| white | 0.10 | 0.029 | -7.630 | 0.000 | 0.05 | 0.17 |  |
| socioeconomic status | 0.89 | 0.036 | -2.860 | 0.004 | 0.82 | 0.96 |  |
| age at puberty | 1.00 | 0.043 | -0.040 | 0.965 | 0.92 | 1.09 |  |
| family size | 1.17 | 0.044 | 4.140 | 0.000 | 1.09 | 1.26 |  |
| birth order | 1.09 | 0.101 | 0.910 | 0.361 | 0.91 | 1.31 |  |
| birth order² | 0.98 | 0.009 | -1.970 | 0.049 | 0.96 | 1.00 |  |
| intercept | 0.00 | 0.000 | -6.820 | 0.000 | 0.00 | 0.00 |  |

|  |  |  |  |  |  |  |  |
| --- | --- | --- | --- | --- | --- | --- | --- |
| **PROGRESSION TO MARRIAGE²** | **O.R.** | **S.E.** | **z** | **P>z** | **95% C.I.** | | **n=4748** |
| *ref: intact family* |  |  |  |  |  |  |  |
| SINGLE DAD | 1.10 | 0.179 | 0.590 | 0.557 | 0.80 | 1.51 |  |
| SINGLE MUM | 1.01 | 0.091 | 0.060 | 0.948 | 0.84 | 1.20 |  |
| DAD + STEPMUM | 1.01 | 0.191 | 0.060 | 0.954 | 0.70 | 1.47 |  |
| MUM + STEPDAD | 1.18 | 0.152 | 1.320 | 0.188 | 0.92 | 1.52 |  |
| FOSTER NON-RELATIVES | 0.66 | 0.169 | -1.630 | 0.103 | 0.40 | 1.09 |  |
| FOSTER RELATIVES | 0.87 | 0.148 | -0.840 | 0.401 | 0.62 | 1.21 |  |
| INSTITUTION | 0.69 | 0.175 | -1.470 | 0.141 | 0.42 | 1.13 |  |
| time | 4.07 | 0.275 | 20.710 | 0.000 | 3.56 | 4.64 |  |
| time² | 0.97 | 0.001 | -17.720 | 0.000 | 0.97 | 0.98 |  |
| age | 1.01 | 0.005 | 1.820 | 0.069 | 1.00 | 1.02 |  |
| year of birth | 1.06 | 0.063 | 0.920 | 0.359 | 0.94 | 1.19 |  |
| white | 0.56 | 0.039 | -8.320 | 0.000 | 0.49 | 0.65 |  |
| socioeconomic status | 0.94 | 0.016 | -3.750 | 0.000 | 0.91 | 0.97 |  |
| age at puberty | 0.93 | 0.016 | -4.020 | 0.000 | 0.90 | 0.97 |  |
| family size | 1.03 | 0.014 | 2.400 | 0.016 | 1.01 | 1.06 |  |
| birth order | 1.01 | 0.037 | 0.380 | 0.703 | 0.94 | 1.09 |  |
| birth order² | 1.00 | 0.004 | -0.880 | 0.381 | 0.99 | 1.00 |  |
| intercept | 0.00 | 0.000 | -23.410 | 0.000 | 0.00 | 0.00 |  |
|  |  |  |  |  |  |  |  |
| **MORE THAN ONE MARRIAGE³** | **O.R.** | **S.E.** | **z** | **P>z** | **95% C.I.** | | **n=1996** |
| *ref: intact family* |  |  |  |  |  |  |  |
| SINGLE DAD | 1.62 | 0.629 | 1.240 | 0.216 | 0.76 | 3.46 |  |
| SINGLE MUM | 1.71 | 0.382 | 2.410 | 0.016 | 1.11 | 2.65 |  |
| DAD + STEPMUM | 0.21 | 0.215 | -1.520 | 0.128 | 0.03 | 1.57 |  |
| MUM + STEPDAD | 2.73 | 0.834 | 3.280 | 0.001 | 1.50 | 4.97 |  |
| FOSTER NON-RELATIVES | 0.34 | 0.360 | -1.020 | 0.309 | 0.04 | 2.74 |  |
| FOSTER RELATIVES | 2.35 | 0.949 | 2.120 | 0.034 | 1.07 | 5.18 |  |
| INSTITUTION | 2.16 | 1.346 | 1.240 | 0.216 | 0.64 | 7.32 |  |
| white | 1.65 | 0.319 | 2.600 | 0.009 | 1.13 | 2.41 |  |
| year of birth | 0.61 | 0.039 | -7.670 | 0.000 | 0.54 | 0.69 |  |
| socioeconomic status | 0.88 | 0.037 | -3.050 | 0.002 | 0.81 | 0.95 |  |
| age at puberty | 0.98 | 0.044 | -0.390 | 0.698 | 0.90 | 1.07 |  |
| family size | 1.05 | 0.034 | 1.430 | 0.154 | 0.98 | 1.12 |  |
| birth order | 1.13 | 0.117 | 1.170 | 0.243 | 0.92 | 1.38 |  |
| birth order² | 0.98 | 0.011 | -1.570 | 0.117 | 0.96 | 1.00 |  |
| intercept | 0.15 | 0.101 | -2.790 | 0.005 | 0.04 | 0.57 |  |

|  |  |  |  |  |  |  |  |
| --- | --- | --- | --- | --- | --- | --- | --- |
| **AGE AT 1ST BIRTH¹** | **Coef.** | **S.E.** | **t** | **P>t** | **95% C.I.** | | **n=799** |
| *ref: intact family* |  |  |  |  |  |  |  |
| SINGLE DAD | 13.97 | 14.930 | 0.940 | 0.350 | -15.33 | 43.27 |  |
| SINGLE MUM | -20.04 | 8.410 | -2.380 | 0.017 | -36.55 | -3.53 |  |
| DAD + STEPMUM | 6.65 | 17.530 | 0.380 | 0.705 | -27.77 | 41.06 |  |
| MUM + STEPDAD | -19.28 | 12.150 | -1.590 | 0.113 | -43.13 | 4.57 |  |
| FOSTER NON-RELATIVES | 17.63 | 39.040 | 0.450 | 0.652 | -59.00 | 94.26 |  |
| FOSTER RELATIVES | 5.75 | 16.110 | 0.360 | 0.721 | -25.87 | 37.36 |  |
| INSTITUTION | -22.62 | 19.670 | -1.150 | 0.251 | -61.23 | 16.00 |  |
| year of birth | -0.97 | 0.200 | -4.770 | 0.000 | -1.37 | -0.57 |  |
| white | 20.78 | 6.610 | 3.140 | 0.002 | 7.80 | 33.76 |  |
| socioeconomic status | 5.60 | 1.300 | 4.320 | 0.000 | 3.06 | 8.14 |  |
| age at puberty | 0.08 | 1.440 | 0.050 | 0.956 | -2.74 | 2.90 |  |
| family size | -4.76 | 1.100 | -4.340 | 0.000 | -6.92 | -2.61 |  |
| birth order | 2.70 | 3.020 | 0.890 | 0.372 | -3.23 | 8.62 |  |
| birth order² | -0.02 | 0.290 | -0.060 | 0.955 | -0.59 | 0.56 |  |
| intercept | 2132.20 | 392.080 | 5.440 | 0.000 | 1362.55 | 2901.86 |  |
|  |  |  |  |  |  |  |  |
| **ANY EXTRAMARITAL SEX³** | **O.R.** | **S.E.** | **z** | **P>z** | **95% C.I.** | |  |
| *ref: intact family* |  |  |  |  |  |  |  |
| SINGLE DAD | 0.78 | 0.244 | -0.800 | 0.424 | 0.42 | 1.44 |  |
| SINGLE MUM | 1.54 | 0.286 | 2.300 | 0.022 | 1.07 | 2.21 |  |
| DAD + STEPMUM | 1.19 | 0.436 | 0.470 | 0.642 | 0.58 | 2.44 |  |
| MUM + STEPDAD | 1.03 | 0.270 | 0.120 | 0.905 | 0.62 | 1.72 |  |
| FOSTER NON-RELATIVES | 0.45 | 0.222 | -1.620 | 0.106 | 0.17 | 1.19 |  |
| FOSTER RELATIVES | 0.83 | 0.275 | -0.570 | 0.566 | 0.43 | 1.59 |  |
| INSTITUTION | 2.52 | 1.374 | 1.700 | 0.089 | 0.87 | 7.34 |  |
| year of birth | 0.84 | 0.042 | -3.540 | 0.000 | 0.76 | 0.92 |  |
| white | 0.23 | 0.034 | -9.880 | 0.000 | 0.17 | 0.30 |  |
| socioeconomic status | 0.92 | 0.029 | -2.690 | 0.007 | 0.86 | 0.98 |  |
| age at puberty | 0.92 | 0.031 | -2.520 | 0.012 | 0.86 | 0.98 |  |
| family size | 1.03 | 0.027 | 1.210 | 0.226 | 0.98 | 1.09 |  |
| birth order | 0.97 | 0.071 | -0.360 | 0.718 | 0.84 | 1.12 |  |
| birth order² | 1.00 | 0.007 | -0.620 | 0.536 | 0.98 | 1.01 |  |
| intercept | 16.28 | 8.588 | 5.290 | 0.000 | 5.79 | 45.78 |  |

|  |  |  |  |  |  |  |  |
| --- | --- | --- | --- | --- | --- | --- | --- |
| **GAMBLING^4^** | **R.R.R.** | **S.E.** | **z** | **P>z** | **95% C.I.** | | **n=4031** |
| **a little** |  |  |  |  |  |  |  |
| *ref: intact family* |  |  |  |  |  |  |  |
| SINGLE DAD | 0.93 | 0.294 | -0.220 | 0.828 | 0.50 | 1.73 |  |
| SINGLE MUM | 1.08 | 0.168 | 0.490 | 0.623 | 0.80 | 1.46 |  |
| DAD + STEPMUM | 0.83 | 0.251 | -0.610 | 0.543 | 0.46 | 1.50 |  |
| MUM + STEPDAD | 0.95 | 0.219 | -0.220 | 0.829 | 0.61 | 1.49 |  |
| FOSTER NON-RELATIVES | 0.77 | 0.332 | -0.610 | 0.543 | 0.33 | 1.79 |  |
| FOSTER RELATIVES | 0.68 | 0.207 | -1.250 | 0.210 | 0.38 | 1.24 |  |
| INSTITUTION | 0.58 | 0.255 | -1.240 | 0.217 | 0.25 | 1.38 |  |
| year of birth | 1.19 | 0.052 | 4.070 | 0.000 | 1.10 | 1.30 |  |
| white | 1.17 | 0.163 | 1.160 | 0.248 | 0.89 | 1.54 |  |
| socioeconomic status | 1.06 | 0.029 | 2.260 | 0.024 | 1.01 | 1.12 |  |
| age at puberty | 0.97 | 0.028 | -0.990 | 0.324 | 0.92 | 1.03 |  |
| family size | 0.98 | 0.024 | -0.770 | 0.439 | 0.94 | 1.03 |  |
| birth order | 1.02 | 0.065 | 0.370 | 0.712 | 0.90 | 1.16 |  |
| birth order² | 1.00 | 0.007 | -0.430 | 0.665 | 0.98 | 1.01 |  |
| intercept | 0.57 | 0.258 | -1.240 | 0.215 | 0.24 | 1.38 |  |
| **more than a little** |  |  |  |  |  |  |  |
| *ref: intact family* |  |  |  |  |  |  |  |
| SINGLE DAD | 2.15 | 0.583 | 2.810 | 0.005 | 1.26 | 3.66 |  |
| SINGLE MUM | 1.64 | 0.248 | 3.260 | 0.001 | 1.22 | 2.20 |  |
| DAD + STEPMUM | 1.08 | 0.345 | 0.230 | 0.819 | 0.57 | 2.02 |  |
| MUM + STEPDAD | 1.61 | 0.343 | 2.240 | 0.025 | 1.06 | 2.45 |  |
| FOSTER NON-RELATIVES | 1.48 | 0.574 | 1.010 | 0.310 | 0.69 | 3.17 |  |
| FOSTER RELATIVES | 1.36 | 0.365 | 1.130 | 0.259 | 0.80 | 2.30 |  |
| INSTITUTION | 1.43 | 0.498 | 1.020 | 0.308 | 0.72 | 2.83 |  |
| year of birth | 1.06 | 0.049 | 1.250 | 0.212 | 0.97 | 1.16 |  |
| white | 0.35 | 0.040 | -9.230 | 0.000 | 0.28 | 0.43 |  |
| socioeconomic status | 0.96 | 0.028 | -1.270 | 0.204 | 0.91 | 1.02 |  |
| age at puberty | 1.01 | 0.031 | 0.210 | 0.833 | 0.95 | 1.07 |  |
| family size | 1.03 | 0.025 | 1.140 | 0.254 | 0.98 | 1.08 |  |
| birth order | 1.00 | 0.067 | -0.040 | 0.968 | 0.87 | 1.14 |  |
| birth order² | 1.00 | 0.007 | -0.480 | 0.632 | 0.98 | 1.01 |  |
| intercept | 0.99 | 0.472 | -0.020 | 0.987 | 0.39 | 2.52 |  |

|  |  |  |  |  |  |  |  |
| --- | --- | --- | --- | --- | --- | --- | --- |
| **USED ILLEGAL DRUGS³** | **O.R.** | **S.E.** | **z** | **P>z** | **95% C.I.** | | **n=4749** |
| *ref: intact family* |  |  |  |  |  |  |  |
| SINGLE DAD | 1.68 | 0.535 | 1.640 | 0.102 | 0.90 | 3.14 |  |
| SINGLE MUM | 1.61 | 0.269 | 2.850 | 0.004 | 1.16 | 2.24 |  |
| DAD + STEPMUM | 1.15 | 0.481 | 0.320 | 0.747 | 0.50 | 2.61 |  |
| MUM + STEPDAD | 2.55 | 0.546 | 4.370 | 0.000 | 1.68 | 3.88 |  |
| FOSTER NON-RELATIVES | 1.58 | 0.706 | 1.020 | 0.308 | 0.66 | 3.80 |  |
| FOSTER RELATIVES | 2.08 | 0.613 | 2.480 | 0.013 | 1.17 | 3.71 |  |
| INSTITUTION | 2.05 | 0.741 | 1.990 | 0.047 | 1.01 | 4.16 |  |
| year of birth | 1.48 | 0.102 | 5.640 | 0.000 | 1.29 | 1.69 |  |
| white | 0.28 | 0.035 | -10.060 | 0.000 | 0.22 | 0.36 |  |
| socioeconomic status | 0.79 | 0.029 | -6.610 | 0.000 | 0.73 | 0.84 |  |
| age at puberty | 0.93 | 0.036 | -1.940 | 0.052 | 0.86 | 1.00 |  |
| family size | 0.96 | 0.030 | -1.180 | 0.237 | 0.91 | 1.02 |  |
| birth order | 1.13 | 0.098 | 1.400 | 0.162 | 0.95 | 1.34 |  |
| birth order² | 0.99 | 0.009 | -0.900 | 0.368 | 0.97 | 1.01 |  |
| intercept | 1.50 | 0.885 | 0.680 | 0.497 | 0.47 | 4.77 |  |
|  |  |  |  |  |  |  |  |
|  |  |  |  |  |  |  |  |

¹ linear regression analyses, ² discrete-time event-history analyses, ³ binary logistic regression analyses, ^4^ multinomial logistic analyses

Coef. = beta coefficient; O.R. = odds ratio; R.R.R = relative risk ratio, S.E. = standard error; C.I. = confidence interval
