# Supplementary material for: A Not-So-Grim Tale: How Childhood Family Structure Influences Reproductive and Risk-Taking Outcomes in a Historical U.S. Population
Source: PLoS One. 2014 Mar 5;9(3):e89539. doi: 10.1371/journal.pone.0089539 (PMC3943735; doi:10.1371/journal.pone.0089539)
Supplement: Table S2 — All results for all models, excluding age at puberty covariate, for (a) women and (b) men. (DOCX) [file pone.0089539.s002.docx]

### Table S2a: All results for all models excluding age at puberty covariate, for women

| **AGE AT 1ST PETTING¹** | **Coef.** | **S.E.** | **t** | **P>t** | **95% C.I.** | | | **n=6700** |
| --- | --- | --- | --- | --- | --- | --- | --- | --- |
| *ref: intact family* |  |  |  |  |  | |  |  |
| SINGLE DAD | -0.63 | 0.269 | -2.350 | 0.019 | -1.16 | | -0.10 |  |
| SINGLE MUM | -0.38 | 0.133 | -2.830 | 0.005 | -0.64 | | -0.12 |  |
| DAD + STEPMUM | -0.36 | 0.248 | -1.460 | 0.146 | -0.85 | | 0.13 |  |
| MUM + STEPDAD | -0.64 | 0.219 | -2.940 | 0.003 | -1.07 | | -0.21 |  |
| FOSTER NON-RELATIVES | -0.49 | 0.378 | -1.300 | 0.195 | -1.23 | | 0.25 |  |
| FOSTER RELATIVES | -0.55 | 0.272 | -2.010 | 0.044 | -1.08 | | -0.01 |  |
| INSTITUTION | 0.32 | 0.422 | 0.750 | 0.454 | -0.51 | | 1.14 |  |
| year of birth | -0.08 | 0.003 | -24.700 | 0.000 | -0.09 | | -0.08 |  |
| white | 1.09 | 0.119 | 9.150 | 0.000 | 0.86 | | 1.33 |  |
| socioeconomic status | 0.13 | 0.025 | 5.050 | 0.000 | 0.08 | | 0.17 |  |
| family size | 0.05 | 0.023 | 2.020 | 0.044 | 0.00 | | 0.09 |  |
| birth order | -0.08 | 0.056 | -1.370 | 0.170 | -0.19 | | 0.03 |  |
| birth order² | 0.00 | 0.006 | -0.680 | 0.499 | -0.02 | | 0.01 |  |
| intercept | 173.11 | 6.420 | 26.970 | 0.000 | 160.53 | | 185.70 |  |
|  |  |  |  |  |  | |  |  |
|  |  |  |  |  |  | |  |  |
| **PROGRESSION TO 1ST SEX²** | **O.R.** | **S.E.** | **z** | **P>z** | **95% C.I.** | | | **n=7039** |
| *ref: intact family* |  |  |  |  |  |  | |  |
| SINGLE DAD | 1.98 | 0.233 | 5.810 | 0.000 | 1.57 | 2.49 | |  |
| SINGLE MUM | 5.28 | 1.255 | 6.990 | 0.000 | 3.31 | 8.41 | |  |
| DAD + STEPMUM | 4.57 | 2.008 | 3.450 | 0.001 | 1.93 | 10.81 | |  |
| MUM + STEPDAD | 2.07 | 0.189 | 7.960 | 0.000 | 1.73 | 2.47 | |  |
| FOSTER CARE* | 2.77 | 0.259 | 10.940 | 0.000 | 2.31 | 3.33 | |  |
| INSTITUTION | 2.37 | 0.393 | 5.180 | 0.000 | 1.71 | 3.28 | |  |
| time | 2.61 | 0.063 | 40.110 | 0.000 | 2.49 | 2.74 | |  |
| time² | 0.98 | 0.001 | -33.920 | 0.000 | 0.98 | 0.98 | |  |
| SINGLE MUM*TIME | 0.93 | 0.012 | -5.590 | 0.000 | 0.91 | 0.96 | |  |
| SINGLE MUM*TIME² | 0.93 | 0.021 | -2.990 | 0.003 | 0.89 | 0.98 | |  |
| year of birth | 0.89 | 0.054 | -1.940 | 0.052 | 0.79 | 1.00 | |  |
| age | 0.98 | 0.005 | -4.210 | 0.000 | 0.97 | 0.99 | |  |
| white | 0.39 | 0.020 | -18.440 | 0.000 | 0.36 | 0.43 | |  |
| socioeconomic status | 0.85 | 0.009 | -14.690 | 0.000 | 0.83 | 0.87 | |  |
| family size | 1.07 | 0.010 | 7.190 | 0.000 | 1.05 | 1.09 | |  |
| birth order | 0.94 | 0.023 | -2.520 | 0.012 | 0.90 | 0.99 | |  |
| birth order² | 1.01 | 0.003 | 2.360 | 0.018 | 1.00 | 1.01 | |  |
| intercept | 0.00 | 0.000 | -38.670 | 0.000 | 0.00 | 0.00 | |  |

|  |  |  |  |  |  |  |  |
| --- | --- | --- | --- | --- | --- | --- | --- |
| **ANY PREMARITAL SEX³** | **O.R.** | **S.E.** | **z** | **P>z** | **95% C.I.** | | **n=4713** |
| *ref: intact family* |  |  |  |  |  |  |  |
| SINGLE DAD | 1.12 | 0.266 | 0.470 | 0.638 | 0.70 | 1.78 |  |
| SINGLE MUM | 1.24 | 0.154 | 1.760 | 0.079 | 0.98 | 1.59 |  |
| DAD + STEPMUM | 0.92 | 0.201 | -0.370 | 0.713 | 0.60 | 1.42 |  |
| MUM + STEPDAD | 1.04 | 0.192 | 0.190 | 0.847 | 0.72 | 1.49 |  |
| FOSTER NON-RELATIVES | 1.68 | 0.647 | 1.350 | 0.175 | 0.79 | 3.58 |  |
| FOSTER RELATIVES | 0.98 | 0.228 | -0.100 | 0.918 | 0.62 | 1.54 |  |
| INSTITUTION | 2.80 | 1.207 | 2.390 | 0.017 | 1.20 | 6.52 |  |
| year of birth | 1.06 | 0.003 | 18.380 | 0.000 | 1.05 | 1.07 |  |
| white | 0.32 | 0.039 | -9.270 | 0.000 | 0.25 | 0.41 |  |
| socioeconomic status | 1.02 | 0.022 | 1.060 | 0.288 | 0.98 | 1.07 |  |
| family size | 1.02 | 0.020 | 1.240 | 0.214 | 0.99 | 1.06 |  |
| birth order | 0.91 | 0.049 | -1.770 | 0.077 | 0.82 | 1.01 |  |
| birth order² | 1.01 | 0.006 | 1.920 | 0.055 | 1.00 | 1.02 |  |
| intercept | 0.00 | 0.000 | -18.090 | 0.000 | 0.00 | 0.00 |  |

|  |  |  |  |  |  |  |  |
| --- | --- | --- | --- | --- | --- | --- | --- |
| **NUMBER OF SEX PARTNERS^4^** | **R.R.R.** | **S.E.** | **z** | **P>z** | **95% C.I.** | | **n=4713** |
| *base outcome: none* |  |  |  |  |  |  |  |
| **1 to 5 partners** |  |  |  |  |  |  |  |
| *ref: intact family* |  |  |  |  |  |  |  |
| SINGLE DAD | 1.11 | 0.273 | 0.430 | 0.666 | 0.69 | 1.80 |  |
| SINGLE MUM | 1.20 | 0.154 | 1.440 | 0.149 | 0.94 | 1.55 |  |
| DAD + STEPMUM | 0.90 | 0.203 | -0.480 | 0.631 | 0.58 | 1.40 |  |
| MUM + STEPDAD | 0.97 | 0.188 | -0.170 | 0.869 | 0.66 | 1.42 |  |
| FOSTER NON-RELATIVES | 1.47 | 0.587 | 0.970 | 0.334 | 0.67 | 3.21 |  |
| FOSTER RELATIVES | 0.92 | 0.224 | -0.330 | 0.738 | 0.57 | 1.48 |  |
| INSTITUTION | 2.43 | 1.085 | 2.000 | 0.046 | 1.02 | 5.83 |  |
| year of birth | 1.06 | 0.003 | 17.100 | 0.000 | 1.05 | 1.06 |  |
| white | 0.34 | 0.043 | -8.520 | 0.000 | 0.26 | 0.44 |  |
| socioeconomic status | 1.04 | 0.023 | 1.840 | 0.065 | 1.00 | 1.09 |  |
| family size | 1.01 | 0.020 | 0.680 | 0.499 | 0.97 | 1.05 |  |
| birth order | 0.93 | 0.051 | -1.340 | 0.181 | 0.83 | 1.03 |  |
| birth order² | 1.01 | 0.006 | 1.740 | 0.081 | 1.00 | 1.02 |  |
| intercept | 0.00 | 0.000 | -16.880 | 0.000 | 0.00 | 0.00 |  |
| **6+ partners** |  |  |  |  |  |  |  |
| *ref: intact family* |  |  |  |  |  |  |  |
| SINGLE DAD | 1.15 | 0.413 | 0.400 | 0.692 | 0.57 | 2.33 |  |
| SINGLE MUM | 1.41 | 0.244 | 2.010 | 0.045 | 1.01 | 1.98 |  |
| DAD + STEPMUM | 1.03 | 0.321 | 0.100 | 0.920 | 0.56 | 1.90 |  |
| MUM + STEPDAD | 1.32 | 0.330 | 1.090 | 0.274 | 0.80 | 2.15 |  |
| FOSTER NON-RELATIVES | 2.54 | 1.170 | 2.020 | 0.043 | 1.03 | 6.26 |  |
| FOSTER RELATIVES | 1.21 | 0.386 | 0.590 | 0.554 | 0.65 | 2.26 |  |
| INSTITUTION | 4.17 | 2.091 | 2.850 | 0.004 | 1.56 | 11.14 |  |
| year of birth | 1.07 | 0.005 | 12.810 | 0.000 | 1.06 | 1.08 |  |
| white | 0.26 | 0.040 | -8.820 | 0.000 | 0.19 | 0.35 |  |
| socioeconomic status | 0.95 | 0.031 | -1.680 | 0.092 | 0.89 | 1.01 |  |
| family size | 1.07 | 0.029 | 2.300 | 0.021 | 1.01 | 1.12 |  |
| birth order | 0.84 | 0.063 | -2.400 | 0.016 | 0.72 | 0.97 |  |
| birth order² | 1.01 | 0.008 | 1.930 | 0.054 | 1.00 | 1.03 |  |
| intercept | 0.00 | 0.000 | -12.740 | 0.000 | 0.00 | 0.00 |  |

| **PROGRESSION TO MARRIAGE²** | **O.R.** | **S.E.** | **z** | **P>z** | **95% C.I.** | | **N=7028** |
| --- | --- | --- | --- | --- | --- | --- | --- |
| *ref: intact family* |  |  |  |  |  |  |  |
| SINGLE DAD | 1.78 | 0.229 | 4.460 | 0.000 | 1.38 | 2.29 |  |
| SINGLE MUM | 1.15 | 0.077 | 2.100 | 0.036 | 1.01 | 1.31 |  |
| DAD + STEPMUM | 1.27 | 0.149 | 2.070 | 0.038 | 1.01 | 1.60 |  |
| MUM + STEPDAD | 1.58 | 0.160 | 4.550 | 0.000 | 1.30 | 1.93 |  |
| FOSTER NON-RELATIVES | 1.57 | 0.307 | 2.330 | 0.020 | 1.07 | 2.31 |  |
| FOSTER RELATIVES | 1.94 | 0.237 | 5.410 | 0.000 | 1.53 | 2.46 |  |
| INSTITUTION | 0.94 | 0.207 | -0.290 | 0.770 | 0.61 | 1.45 |  |
| time | 3.28 | 0.136 | 28.720 | 0.000 | 3.03 | 3.56 |  |
| time² | 0.98 | 0.001 | -25.090 | 0.000 | 0.97 | 0.98 |  |
| age | 0.98 | 0.006 | -3.320 | 0.001 | 0.97 | 0.99 |  |
| year of birth | 0.74 | 0.051 | -4.280 | 0.000 | 0.65 | 0.85 |  |
| white | 0.73 | 0.042 | -5.530 | 0.000 | 0.65 | 0.81 |  |
| socioeconomic status | 0.86 | 0.011 | -12.350 | 0.000 | 0.84 | 0.88 |  |
| family size | 1.04 | 0.011 | 3.540 | 0.000 | 1.02 | 1.06 |  |
| birth order | 0.97 | 0.027 | -1.260 | 0.207 | 0.91 | 1.02 |  |
| birth order² | 1.00 | 0.003 | 1.140 | 0.255 | 1.00 | 1.01 |  |
| intercept | 0.00 | 0.000 | -31.860 | 0.000 | 0.00 | 0.00 |  |
|  |  |  |  |  |  |  |  |
| **MORE THAN ONE MARRIAGE³** | **O.R.** | **S.E.** | **z** | **P>z** | **95% C.I.** | | **N=3509** |
| *ref: intact family* |  |  |  |  |  |  |  |
| SINGLE DAD | 1.21 | 0.354 | 0.660 | 0.512 | 0.68 | 2.15 |  |
| SINGLE MUM | 1.26 | 0.194 | 1.520 | 0.128 | 0.93 | 1.71 |  |
| DAD + STEPMUM | 1.61 | 0.422 | 1.810 | 0.071 | 0.96 | 2.69 |  |
| MUM + STEPDAD | 1.50 | 0.342 | 1.790 | 0.074 | 0.96 | 2.35 |  |
| FOSTER NON-RELATIVES | 2.34 | 1.018 | 1.960 | 0.050 | 1.00 | 5.49 |  |
| FOSTER RELATIVES | 2.02 | 0.516 | 2.770 | 0.006 | 1.23 | 3.33 |  |
| INSTITUTION | 1.21 | 0.600 | 0.380 | 0.702 | 0.46 | 3.20 |  |
| white | 0.92 | 0.126 | -0.590 | 0.556 | 0.71 | 1.21 |  |
| year of birth | 0.61 | 0.026 | -11.580 | 0.000 | 0.56 | 0.67 |  |
| socioeconomic status | 0.86 | 0.024 | -5.330 | 0.000 | 0.81 | 0.91 |  |
| family size | 0.99 | 0.025 | -0.380 | 0.704 | 0.94 | 1.04 |  |
| birth order | 0.96 | 0.060 | -0.670 | 0.506 | 0.85 | 1.08 |  |
| birth order² | 1.01 | 0.006 | 1.780 | 0.075 | 1.00 | 1.02 |  |
| intercept | 0.35 | 0.068 | -5.380 | 0.000 | 0.24 | 0.51 |  |
|  |  |  |  |  |  |  |  |

| **AGE AT 1ST BIRTH¹** | **Coef.** | **S.E.** | **t** | **P>t** | **95% C.I.** | | **N=1239** |
| --- | --- | --- | --- | --- | --- | --- | --- |
| *ref: intact family* |  |  |  |  |  |  |  |
| SINGLE DAD | -41.51 | 12.626 | -3.290 | 0.001 | -66.28 | -16.74 |  |
| SINGLE MUM | -10.02 | 6.440 | -1.560 | 0.120 | -22.65 | 2.62 |  |
| DAD + STEPMUM | -9.74 | 11.439 | -0.850 | 0.395 | -32.18 | 12.70 |  |
| MUM + STEPDAD | -42.85 | 10.154 | -4.220 | 0.000 | -62.77 | -22.93 |  |
| FOSTER NON-RELATIVES | -78.54 | 29.424 | -2.670 | 0.008 | -136.27 | -20.81 |  |
| FOSTER RELATIVES | -20.02 | 12.998 | -1.540 | 0.124 | -45.52 | 5.48 |  |
| INSTITUTION | -13.03 | 29.301 | -0.440 | 0.657 | -70.52 | 44.45 |  |
| year of birth | -0.27 | 0.175 | -1.540 | 0.124 | -0.61 | 0.07 |  |
| white | 36.28 | 6.681 | 5.430 | 0.000 | 23.17 | 49.39 |  |
| socioeconomic status | 7.01 | 1.056 | 6.640 | 0.000 | 4.94 | 9.08 |  |
| family size | -3.68 | 0.880 | -4.180 | 0.000 | -5.41 | -1.96 |  |
| birth order | 0.68 | 2.663 | 0.260 | 0.798 | -4.54 | 5.91 |  |
| birth order² | -0.16 | 0.289 | -0.550 | 0.585 | -0.73 | 0.41 |  |
| intercept | 752.05 | 332.940 | 2.260 | 0.024 | 98.85 | 1405.24 |  |
|  |  |  |  |  |  |  |  |
| **ANY EXTRAMARITAL SEX³** | **O.R.** | **S.E.** | **z** | **P>z** | **95% C.I.** | | **n=3588** |
| *ref: intact family* |  |  |  |  |  |  |  |
| SINGLE DAD | 0.92 | 0.249501 | -0.29 | 0.769 | 0.54 | 1.57 |  |
| SINGLE MUM | 1.13 | 0.15464 | 0.87 | 0.382 | 0.86 | 1.48 |  |
| DAD + STEPMUM | 0.62 | 0.174282 | -1.7 | 0.088 | 0.36 | 1.07 |  |
| MUM + STEPDAD | 1.01 | 0.212573 | 0.03 | 0.975 | 0.67 | 1.52 |  |
| FOSTER NON-REL | 1.15 | 0.462113 | 0.35 | 0.726 | 0.52 | 2.53 |  |
| FOSTER REL | 1.55 | 0.359054 | 1.89 | 0.059 | 0.98 | 2.44 |  |
| INSTITUTION | 1.35 | 0.617479 | 0.66 | 0.510 | 0.55 | 3.31 |  |
| year of birth | 1.00 | 0.003577 | -0.22 | 0.826 | 0.99 | 1.01 |  |
| white | 0.74 | 0.088859 | -2.49 | 0.013 | 0.59 | 0.94 |  |
| socioeconomic status | 1.08 | 0.027156 | 3.02 | 0.003 | 1.03 | 1.13 |  |
| family size | 0.97 | 0.022256 | -1.3 | 0.193 | 0.93 | 1.02 |  |
| birth order | 0.99 | 0.058394 | -0.22 | 0.829 | 0.88 | 1.11 |  |
| birth order² | 1.00 | 0.006006 | 0.16 | 0.870 | 0.99 | 1.01 |  |
| intercept | 1.65 | 11.33435 | 0.07 | 0.942 | 0.00 | 1.14E+06 |  |

| **GAMBLING^4^** | **R.R.R.** | **S.E.** | **z** | **P>z** | **95% C.I.** | | **n=6682** |
| --- | --- | --- | --- | --- | --- | --- | --- |
| **a little** |  |  |  |  |  |  |  |
| *ref: intact family* |  |  |  |  |  |  |  |
| SINGLE DAD | 0.79 | 0.210 | -0.900 | 0.370 | 0.47 | 1.33 |  |
| SINGLE MUM | 1.08 | 0.130 | 0.670 | 0.506 | 0.86 | 1.37 |  |
| DAD + STEPMUM | 1.21 | 0.245 | 0.920 | 0.355 | 0.81 | 1.80 |  |
| MUM + STEPDAD | 0.86 | 0.187 | -0.710 | 0.477 | 0.56 | 1.31 |  |
| FOSTER NON-RELATIVES | 0.62 | 0.256 | -1.150 | 0.249 | 0.28 | 1.39 |  |
| FOSTER RELATIVES | 0.86 | 0.238 | -0.540 | 0.587 | 0.50 | 1.48 |  |
| INSTITUTION | 1.35 | 0.498 | 0.800 | 0.422 | 0.65 | 2.78 |  |
| year of birth | 0.89 | 0.027 | -3.890 | 0.000 | 0.84 | 0.94 |  |
| white | 0.89 | 0.101 | -1.010 | 0.315 | 0.71 | 1.11 |  |
| socioeconomic status | 1.19 | 0.027 | 7.660 | 0.000 | 1.14 | 1.25 |  |
| family size | 0.94 | 0.020 | -2.980 | 0.003 | 0.90 | 0.98 |  |
| birth order | 1.05 | 0.057 | 0.900 | 0.366 | 0.94 | 1.17 |  |
| birth order² | 1.00 | 0.006 | -0.270 | 0.789 | 0.99 | 1.01 |  |
| intercept | 0.15 | 0.025 | -11.420 | 0.000 | 0.11 | 0.21 |  |
| **more than a little** |  |  |  |  |  |  |  |
| *ref: intact family* |  |  |  |  |  |  |  |
| SINGLE DAD | 1.49 | 0.481 | 1.250 | 0.213 | 0.79 | 2.81 |  |
| SINGLE MUM | 1.39 | 0.232 | 1.960 | 0.049 | 1.00 | 1.92 |  |
| DAD + STEPMUM | 0.37 | 0.193 | -1.910 | 0.057 | 0.14 | 1.03 |  |
| MUM + STEPDAD | 2.67 | 0.578 | 4.550 | 0.000 | 1.75 | 4.09 |  |
| FOSTER NON-RELATIVES | 1.06 | 0.566 | 0.110 | 0.909 | 0.37 | 3.02 |  |
| FOSTER RELATIVES | 3.64 | 0.887 | 5.310 | 0.000 | 2.26 | 5.87 |  |
| INSTITUTION | 2.30 | 1.051 | 1.830 | 0.068 | 0.94 | 5.63 |  |
| year of birth | 0.84 | 0.039 | -3.680 | 0.000 | 0.77 | 0.92 |  |
| white | 0.37 | 0.048 | -7.700 | 0.000 | 0.29 | 0.48 |  |
| socioeconomic status | 1.01 | 0.035 | 0.250 | 0.802 | 0.94 | 1.08 |  |
| family size | 0.97 | 0.030 | -0.920 | 0.359 | 0.91 | 1.03 |  |
| birth order | 1.08 | 0.076 | 1.120 | 0.262 | 0.94 | 1.24 |  |
| birth order² | 1.00 | 0.006 | 0.290 | 0.776 | 0.99 | 1.01 |  |
| intercept | 0.17 | 0.036 | -8.260 | 0.000 | 0.11 | 0.25 |  |

| **USED ILLEGAL DRUGS³** | **O.R.** | **S.E.** | **z** | **P>z** | **95% C.I.** | | **N=6600** |
| --- | --- | --- | --- | --- | --- | --- | --- |
| *ref: intact family* |  |  |  |  |  |  |  |
| SINGLE DAD | 0.96 | 0.510 | -0.070 | 0.945 | 0.34 | 2.72 |  |
| SINGLE MUM | 1.87 | 0.368 | 3.180 | 0.001 | 1.27 | 2.75 |  |
| DAD + STEPMUM | 1.77 | 0.652 | 1.560 | 0.120 | 0.86 | 3.65 |  |
| MUM + STEPDAD | 2.82 | 0.766 | 3.820 | 0.000 | 1.66 | 4.81 |  |
| FOSTER NON-RELATIVES | 4.70 | 1.862 | 3.900 | 0.000 | 2.16 | 10.21 |  |
| FOSTER RELATIVES | 3.99 | 1.138 | 4.840 | 0.000 | 2.28 | 6.97 |  |
| INSTITUTION | 2.52 | 1.257 | 1.860 | 0.063 | 0.95 | 6.70 |  |
| year of birth | 1.00 | 0.068 | 0.000 | 0.997 | 0.87 | 1.14 |  |
| white | 0.32 | 0.051 | -7.220 | 0.000 | 0.24 | 0.44 |  |
| socioeconomic status | 0.80 | 0.036 | -5.050 | 0.000 | 0.73 | 0.87 |  |
| family size | 0.86 | 0.042 | -3.050 | 0.002 | 0.78 | 0.95 |  |
| birth order | 1.08 | 0.102 | 0.860 | 0.390 | 0.90 | 1.30 |  |
| birth order² | 1.01 | 0.007 | 1.450 | 0.146 | 1.00 | 1.03 |  |
| intercept | 0.22 | 0.060 | -5.580 | 0.000 | 0.13 | 0.38 |  |
|  |  |  |  |  |  |  |  |

### Table S2b: All results for all models excluding age at puberty covariate, for men

| **AGE AT 1ST PETTING¹** | **Coef.** | **S.E.** | **t** | **P>t** | **95% C.I.** | | **n=8331** |
| --- | --- | --- | --- | --- | --- | --- | --- |
| *ref: intact family* |  |  |  |  |  |  |  |
| SINGLE DAD | -0.24 | 0.240 | -1.000 | 0.319 | -0.71 | 0.23 |  |
| SINGLE MUM | -0.05 | 0.127 | -0.380 | 0.702 | -0.30 | 0.20 |  |
| DAD + STEPMUM | -0.16 | 0.263 | -0.590 | 0.555 | -0.67 | 0.36 |  |
| MUM + STEPDAD | -0.42 | 0.190 | -2.220 | 0.026 | -0.79 | -0.05 |  |
| FOSTER NON-RELATIVES | -0.36 | 0.333 | -1.070 | 0.284 | -1.01 | 0.30 |  |
| FOSTER RELATIVES | -0.34 | 0.227 | -1.500 | 0.133 | -0.79 | 0.10 |  |
| INSTITUTION | 0.61 | 0.320 | 1.900 | 0.057 | -0.02 | 1.24 |  |
| year of birth | -0.06 | 0.003 | -20.110 | 0.000 | -0.06 | -0.05 |  |
| white | 1.22 | 0.102 | 11.950 | 0.000 | 1.02 | 1.42 |  |
| socioeconomic status | 0.02 | 0.023 | 1.020 | 0.306 | -0.02 | 0.07 |  |
| family size | 0.04 | 0.019 | 1.840 | 0.065 | 0.00 | 0.07 |  |
| birth order | -0.02 | 0.051 | -0.460 | 0.646 | -0.12 | 0.08 |  |
| birth order² | -0.01 | 0.005 | -1.170 | 0.241 | -0.02 | 0.00 |  |
| intercept | 127.64 | 5.633 | 22.660 | 0.000 | 116.60 | 138.68 |  |
|  |  |  |  |  |  |  |  |
| **PROGRESSION TO 1ST SEX²** | **O.R.** | **S.E.** | **z** | **P>z** | **95% C.I.** | | **n=8668** |
| *ref: intact family* |  |  |  |  |  |  |  |
| SINGLE DAD | 1.33 | 0.124 | 3.060 | 0.002 | 1.11 | 1.60 |  |
| SINGLE MUM | 1.98 | 0.214 | 6.340 | 0.000 | 1.60 | 2.45 |  |
| DAD + STEPMUM | 1.23 | 0.129 | 2.010 | 0.044 | 1.01 | 1.52 |  |
| MUM + STEPDAD | 1.39 | 0.104 | 4.420 | 0.000 | 1.20 | 1.61 |  |
| FOSTER NON-RELATIVES | 1.34 | 0.173 | 2.250 | 0.024 | 1.04 | 1.72 |  |
| FOSTER RELATIVES | 1.20 | 0.108 | 1.980 | 0.048 | 1.01 | 1.43 |  |
| INSTITUTION | 1.01 | 0.124 | 0.100 | 0.917 | 0.80 | 1.29 |  |
| time | 2.51 | 0.054 | 42.510 | 0.000 | 2.40 | 2.61 |  |
| time² | 0.98 | 0.001 | -36.390 | 0.000 | 0.98 | 0.98 |  |
| SINGLE MUM*TIME² | 0.99 | 0.000 | -5.700 | 0.000 | 0.99 | 0.99 |  |
| year of birth | 1.02 | 0.032 | 0.600 | 0.551 | 0.96 | 1.08 |  |
| age | 1.00 | 0.003 | 0.410 | 0.685 | 0.99 | 1.01 |  |
| white | 0.37 | 0.015 | -24.380 | 0.000 | 0.34 | 0.40 |  |
| socioeconomic status | 0.90 | 0.008 | -11.040 | 0.000 | 0.89 | 0.920 |  |
| family size | 1.05 | 0.008 | 6.200 | 0.000 | 1.03 | 1.06 |  |
| birth order | 1.02 | 0.021 | 0.910 | 0.365 | 0.98 | 1.06 |  |
| birth order² | 1.00 | 0.002 | -0.880 | 0.381 | 0.99 | 1.01 |  |
| intercept | 0.00 | 0.000 | -46.620 | 0.000 | 1.82E-05 | 4.39E-05 |  |
|  |  |  |  |  |  |  |  |

| **ANY PREMARITAL SEX³** | **O.R.** | **S.E.** | **z** | **P>z** | **95% C.I.** | | **n=6736** |
| --- | --- | --- | --- | --- | --- | --- | --- |
| *ref: intact family* |  |  |  |  |  |  |  |
| SINGLE DAD | 1.42 | 0.431 | 1.150 | 0.251 | 0.78 | 2.57 |  |
| SINGLE MUM | 1.22 | 0.196 | 1.220 | 0.224 | 0.89 | 1.67 |  |
| DAD + STEPMUM | 1.26 | 0.415 | 0.690 | 0.490 | 0.66 | 2.40 |  |
| MUM + STEPDAD | 2.27 | 0.720 | 2.580 | 0.010 | 1.22 | 4.23 |  |
| FOSTER NON-RELATIVES | 1.14 | 0.430 | 0.340 | 0.731 | 0.54 | 2.39 |  |
| FOSTER RELATIVES | 2.00 | 0.710 | 1.950 | 0.052 | 0.99 | 4.01 |  |
| INSTITUTION | 1.50 | 0.633 | 0.970 | 0.333 | 0.66 | 3.43 |  |
| year of birth | 1.04 | 0.003 | 14.130 | 0.000 | 1.04 | 1.05 |  |
| white | 0.13 | 0.031 | -8.580 | 0.000 | 0.08 | 0.21 |  |
| socioeconomic status | 0.97 | 0.025 | -1.260 | 0.206 | 0.92 | 1.02 |  |
| family size | 1.06 | 0.025 | 2.500 | 0.012 | 1.01 | 1.11 |  |
| birth order | 1.00 | 0.063 | 0.000 | 0.998 | 0.88 | 1.13 |  |
| birth order² | 1.00 | 0.007 | 0.160 | 0.872 | 0.99 | 1.01 |  |
| intercept | 0.00 | 0.000 | -13.510 | 0.000 | 0.00 | 0.00 |  |
|  |  |  |  |  |  |  |  |

| **NUMBER OF SEX PARTNERS^4^** | **R.R.R.** | **S.E.** | **z** | **P>z** | **95% C.I.** | | **n=6736** |
| --- | --- | --- | --- | --- | --- | --- | --- |
| *base outcome: none* |  |  |  |  |  |  |  |
| **1 to 10 partners** |  |  |  |  |  |  |  |
| *ref: intact family* |  |  |  |  |  |  |  |
| SINGLE DAD | 1.23 | 0.386 | 0.650 | 0.516 | 0.66 | 2.27 |  |
| SINGLE MUM | 1.10 | 0.183 | 0.570 | 0.567 | 0.79 | 1.52 |  |
| DAD + STEPMUM | 1.15 | 0.392 | 0.410 | 0.684 | 0.59 | 2.24 |  |
| MUM + STEPDAD | 2.08 | 0.670 | 2.270 | 0.023 | 1.10 | 3.91 |  |
| FOSTER NON-RELATIVES | 0.93 | 0.368 | -0.190 | 0.850 | 0.43 | 2.02 |  |
| FOSTER RELATIVES | 1.60 | 0.586 | 1.300 | 0.195 | 0.78 | 3.28 |  |
| INSTITUTION | 1.51 | 0.649 | 0.950 | 0.341 | 0.65 | 3.51 |  |
| year of birth | 1.04 | 0.003 | 13.800 | 0.000 | 1.04 | 1.05 |  |
| white | 0.21 | 0.051 | -6.490 | 0.000 | 0.13 | 0.34 |  |
| socioeconomic status | 1.00 | 0.026 | 0.160 | 0.875 | 0.95 | 1.06 |  |
| family size | 1.05 | 0.025 | 2.060 | 0.039 | 1.00 | 1.10 |  |
| birth order | 0.97 | 0.063 | -0.400 | 0.686 | 0.86 | 1.11 |  |
| birth order² | 1.00 | 0.007 | 0.480 | 0.629 | 0.99 | 1.02 |  |
| intercept | 0.00 | 0.000 | -13.350 | 0.000 | 0.00 | 0.00 |  |
| **10+ partners** |  |  |  |  |  |  |  |
| *ref: intact family* |  |  |  |  |  |  |  |
| SINGLE DAD | 1.83 | 0.595 | 1.850 | 0.064 | 0.97 | 3.46 |  |
| SINGLE MUM | 1.45 | 0.251 | 2.140 | 0.033 | 1.03 | 2.03 |  |
| DAD + STEPMUM | 1.48 | 0.531 | 1.090 | 0.274 | 0.73 | 2.99 |  |
| MUM + STEPDAD | 2.67 | 0.881 | 2.970 | 0.003 | 1.40 | 5.10 |  |
| FOSTER NON-RELATIVES | 1.57 | 0.637 | 1.110 | 0.267 | 0.71 | 3.47 |  |
| FOSTER RELATIVES | 2.82 | 1.042 | 2.810 | 0.005 | 1.37 | 5.82 |  |
| INSTITUTION | 1.50 | 0.676 | 0.900 | 0.366 | 0.62 | 3.63 |  |
| year of birth | 1.04 | 0.004 | 11.880 | 0.000 | 1.03 | 1.05 |  |
| white | 0.08 | 0.019 | -10.570 | 0.000 | 0.05 | 0.13 |  |
| socioeconomic status | 0.90 | 0.026 | -3.670 | 0.000 | 0.85 | 0.95 |  |
| family size | 1.08 | 0.027 | 2.960 | 0.003 | 1.03 | 1.13 |  |
| birth order | 1.06 | 0.073 | 0.790 | 0.429 | 0.92 | 1.21 |  |
| birth order² | 1.00 | 0.007 | -0.510 | 0.613 | 0.98 | 1.01 |  |
| intercept | 0.00 | 0.000 | -11.420 | 0.000 | 0.00 | 0.00 |  |

| **PROGRESSION TO MARRIAGE²** | **O.R.** | **S.E.** | **z** | **P>z** | **95% C.I.** | | **n=8676** |
| --- | --- | --- | --- | --- | --- | --- | --- |
| *ref: intact family* |  |  |  |  |  |  |  |
| SINGLE DAD | 1.21 | 0.140 | 1.680 | 0.093 | 0.97 | 1.52 |  |
| SINGLE MUM | 1.03 | 0.065 | 0.410 | 0.682 | 0.91 | 1.16 |  |
| DAD + STEPMUM | 0.90 | 0.126 | -0.780 | 0.435 | 0.68 | 1.18 |  |
| MUM + STEPDAD | 1.25 | 0.117 | 2.420 | 0.016 | 1.04 | 1.50 |  |
| FOSTER NON-RELATIVES | 0.70 | 0.122 | -2.020 | 0.043 | 0.50 | 0.99 |  |
| FOSTER RELATIVES | 1.07 | 0.119 | 0.650 | 0.517 | 0.87 | 1.33 |  |
| INSTITUTION | 0.58 | 0.105 | -3.010 | 0.003 | 0.40 | 0.83 |  |
| time | 4.13 | 0.199 | 29.380 | 0.000 | 3.76 | 4.54 |  |
| time² | 0.97 | 0.001 | -25.410 | 0.000 | 0.97 | 0.98 |  |
| age | 1.00 | 0.004 | 0.920 | 0.357 | 1.00 | 1.01 |  |
| year of birth | 0.99 | 0.040 | -0.180 | 0.855 | 0.92 | 1.07 |  |
| white | 0.65 | 0.031 | -8.980 | 0.000 | 0.59 | 0.71 |  |
| socioeconomic status | 0.94 | 0.011 | -5.790 | 0.000 | 0.91 | 0.96 |  |
| family size | 1.04 | 0.010 | 3.990 | 0.000 | 1.02 | 1.06 |  |
| birth order | 0.97 | 0.024 | -1.260 | 0.206 | 0.92 | 1.02 |  |
| birth order² | 1.00 | 0.002 | 0.350 | 0.730 | 1.00 | 1.01 |  |
| intercept | 0.00 | 0.000 | -36.180 | 0.000 | 0.00 | 0.00 |  |
|  |  |  |  |  |  |  |  |
| **MORE THAN ONE MARRIAGE³** | **O.R.** | **S.E.** | **z** | **P>z** | **95% C.I.** | | **n=3997** |
| *ref: intact family* |  |  |  |  |  |  |  |
| SINGLE DAD | 1.55 | 0.406 | 1.680 | 0.093 | 0.93 | 2.59 |  |
| SINGLE MUM | 1.55 | 0.234 | 2.930 | 0.003 | 1.16 | 2.09 |  |
| DAD + STEPMUM | 0.83 | 0.315 | -0.480 | 0.628 | 0.40 | 1.75 |  |
| MUM + STEPDAD | 2.02 | 0.444 | 3.220 | 0.001 | 1.32 | 3.11 |  |
| FOSTER NON-RELATIVES | 0.93 | 0.423 | -0.150 | 0.880 | 0.38 | 2.27 |  |
| FOSTER RELATIVES | 2.26 | 0.564 | 3.270 | 0.001 | 1.39 | 3.69 |  |
| INSTITUTION | 0.98 | 0.495 | -0.040 | 0.967 | 0.36 | 2.64 |  |
| white | 1.18 | 0.144 | 1.390 | 0.165 | 0.93 | 1.50 |  |
| year of birth | 0.63 | 0.023 | -12.530 | 0.000 | 0.58 | 0.67 |  |
| socioeconomic status | 0.89 | 0.025 | -4.030 | 0.000 | 0.85 | 0.94 |  |
| family size | 1.07 | 0.023 | 3.090 | 0.002 | 1.02 | 1.11 |  |
| birth order | 0.96 | 0.059 | -0.670 | 0.504 | 0.85 | 1.08 |  |
| birth order² | 1.00 | 0.006 | -0.020 | 0.982 | 0.99 | 1.01 |  |
| intercept | 0.19 | 0.036 | -8.650 | 0.000 | 0.13 | 0.28 |  |

| **AGE AT 1ST BIRTH¹** | **Coef.** | **S.E.** | **t** | **P>t** | **95% C.I.** | | **n=1683** |
| --- | --- | --- | --- | --- | --- | --- | --- |
| *ref: intact family* |  |  |  |  |  |  |  |
| SINGLE DAD | 8.19 | 10.038 | 0.820 | 0.415 | -11.50 | 27.88 |  |
| SINGLE MUM | -9.99 | 5.880 | -1.700 | 0.089 | -21.52 | 1.54 |  |
| DAD + STEPMUM | 11.30 | 13.109 | 0.860 | 0.389 | -14.42 | 37.01 |  |
| MUM + STEPDAD | -26.11 | 8.785 | -2.970 | 0.003 | -43.34 | -8.88 |  |
| FOSTER NON-RELATIVES | -14.60 | 18.985 | -0.770 | 0.442 | -51.83 | 22.64 |  |
| FOSTER RELATIVES | -13.92 | 10.600 | -1.310 | 0.189 | -34.71 | 6.87 |  |
| INSTITUTION | -18.61 | 15.329 | -1.210 | 0.225 | -48.68 | 11.46 |  |
| year of birth | -0.75 | 0.125 | -5.990 | 0.000 | -1.00 | -0.51 |  |
| white | 20.34 | 4.541 | 4.480 | 0.000 | 11.43 | 29.24 |  |
| socioeconomic status | 5.23 | 0.912 | 5.730 | 0.000 | 3.44 | 7.02 |  |
| family size | -3.84 | 0.767 | -5.000 | 0.000 | -5.34 | -2.33 |  |
| birth order | 1.53 | 2.064 | 0.740 | 0.458 | -2.52 | 5.58 |  |
| birth order² | 0.04 | 0.202 | 0.190 | 0.852 | -0.36 | 0.43 |  |
| intercept | 1721.96 | 238.579 | 7.220 | 0.000 | 1254.01 | 2189.90 |  |
|  |  |  |  |  |  |  |  |
| **ANY EXTRAMARITAL SEX³** | **O.R.** | **S.E.** | **z** | **P>z** | **95% C.I.** | | **n=4182** |
| *ref: intact family* |  |  |  |  |  |  |  |
| SINGLE DAD | 0.78 | 0.173 | -1.11 | 0.268 | 0.51 | 1.21 |  |
| SINGLE MUM | 1.31 | 0.164 | 2.15 | 0.031 | 1.02 | 1.67 |  |
| DAD + STEPMUM | 1.22 | 0.326 | 0.76 | 0.450 | 0.73 | 2.06 |  |
| MUM + STEPDAD | 1.01 | 0.187 | 0.03 | 0.974 | 0.70 | 1.45 |  |
| FOSTER NON-REL | 0.71 | 0.230 | -1.06 | 0.288 | 0.37 | 1.34 |  |
| FOSTER REL | 0.89 | 0.193 | -0.55 | 0.583 | 0.58 | 1.36 |  |
| INSTITUTION | 1.61 | 0.571 | 1.35 | 0.177 | 0.81 | 3.23 |  |
| year of birth | 1.00 | 0.003 | -0.22 | 0.826 | 0.99 | 1.00 |  |
| white | 0.24 | 0.025 | -13.81 | 0.000 | 0.20 | 0.30 |  |
| socioeconomic status | 0.98 | 0.021 | -1.04 | 0.296 | 0.94 | 1.02 |  |
| family size | 1.05 | 0.019 | 2.48 | 0.013 | 1.01 | 1.08 |  |
| birth order | 0.98 | 0.047 | -0.5 | 0.619 | 0.89 | 1.07 |  |
| birth order² | 1.00 | 0.005 | -0.26 | 0.797 | 0.99 | 1.01 |  |
| intercept | 10.86 | 55.983 | 0.46 | 0.644 | 0.00 | 2.66E+05 |  |

| **GAMBLING^4^** | **R.R.R.** | **S.E.** | **z** | **P>z** | **95% C.I.** | | **n=7262** |
| --- | --- | --- | --- | --- | --- | --- | --- |
| **a little** |  |  |  |  |  |  |  |
| *ref: intact family* |  |  |  |  |  |  |  |
| SINGLE DAD | 1.08 | 0.242 | 0.350 | 0.725 | 0.70 | 1.68 |  |
| SINGLE MUM | 1.10 | 0.124 | 0.880 | 0.379 | 0.89 | 1.38 |  |
| DAD + STEPMUM | 1.09 | 0.250 | 0.390 | 0.695 | 0.70 | 1.71 |  |
| MUM + STEPDAD | 0.94 | 0.158 | -0.360 | 0.716 | 0.68 | 1.31 |  |
| FOSTER NON-RELATIVES | 0.99 | 0.288 | -0.040 | 0.972 | 0.56 | 1.75 |  |
| FOSTER RELATIVES | 0.88 | 0.182 | -0.620 | 0.536 | 0.59 | 1.32 |  |
| INSTITUTION | 0.64 | 0.194 | -1.480 | 0.139 | 0.35 | 1.16 |  |
| year of birth | 1.23 | 0.036 | 7.160 | 0.000 | 1.16 | 1.31 |  |
| white | 1.09 | 0.107 | 0.860 | 0.391 | 0.90 | 1.32 |  |
| socioeconomic status | 1.07 | 0.021 | 3.460 | 0.001 | 1.03 | 1.11 |  |
| family size | 0.96 | 0.017 | -2.540 | 0.011 | 0.92 | 0.99 |  |
| birth order | 1.02 | 0.045 | 0.340 | 0.731 | 0.93 | 1.11 |  |
| birth order² | 1.00 | 0.005 | 0.940 | 0.345 | 1.00 | 1.01 |  |
| intercept | 0.41 | 0.058 | -6.280 | 0.000 | 0.31 | 0.54 |  |
| **more than a little** |  |  |  |  |  |  |  |
| *ref: intact family* |  |  |  |  |  |  |  |
| SINGLE DAD | 1.82 | 0.373 | 2.910 | 0.004 | 1.21 | 2.72 |  |
| SINGLE MUM | 1.34 | 0.149 | 2.590 | 0.010 | 1.07 | 1.66 |  |
| DAD + STEPMUM | 1.24 | 0.299 | 0.890 | 0.373 | 0.77 | 1.99 |  |
| MUM + STEPDAD | 1.19 | 0.195 | 1.090 | 0.277 | 0.87 | 1.65 |  |
| FOSTER NON-RELATIVES | 1.33 | 0.380 | 1.010 | 0.314 | 0.76 | 2.33 |  |
| FOSTER RELATIVES | 1.43 | 0.269 | 1.880 | 0.059 | 0.99 | 2.07 |  |
| INSTITUTION | 1.08 | 0.284 | 0.280 | 0.781 | 0.64 | 1.80 |  |
| year of birth | 1.12 | 0.033 | 3.770 | 0.000 | 1.06 | 1.19 |  |
| white | 0.38 | 0.031 | -11.680 | 0.000 | 0.32 | 0.45 |  |
| socioeconomic status | 0.96 | 0.020 | -1.990 | 0.046 | 0.92 | 1.00 |  |
| family size | 1.02 | 0.018 | 0.920 | 0.359 | 0.98 | 1.05 |  |
| birth order | 1.00 | 0.047 | 0.030 | 0.975 | 0.91 | 1.10 |  |
| birth order² | 1.00 | 0.005 | 0.270 | 0.786 | 0.99 | 1.01 |  |
| intercept | 1.05 | 0.145 | 0.380 | 0.707 | 0.80 | 1.38 |  |

| **USED ILLEGAL DRUGS³** | **O.R.** | **S.E.** | **z** | **P>z** | **95% C.I.** | | **N=8677** |
| --- | --- | --- | --- | --- | --- | --- | --- |
| *ref: intact family* |  |  |  |  |  |  |  |
| SINGLE DAD | 1.29 | 0.329 | 0.990 | 0.321 | 0.78 | 2.13 |  |
| SINGLE MUM | 1.75 | 0.210 | 4.690 | 0.000 | 1.39 | 2.22 |  |
| DAD + STEPMUM | 1.53 | 0.431 | 1.520 | 0.130 | 0.88 | 2.66 |  |
| MUM + STEPDAD | 2.31 | 0.372 | 5.180 | 0.000 | 1.68 | 3.17 |  |
| FOSTER NON-RELATIVES | 2.06 | 0.613 | 2.440 | 0.015 | 1.15 | 3.69 |  |
| FOSTER RELATIVES | 2.67 | 0.509 | 5.160 | 0.000 | 1.84 | 3.88 |  |
| INSTITUTION | 2.86 | 0.704 | 4.270 | 0.000 | 1.76 | 4.63 |  |
| year of birth | 1.55 | 0.069 | 9.790 | 0.000 | 1.42 | 1.69 |  |
| white | 0.29 | 0.026 | -13.960 | 0.000 | 0.24 | 0.34 |  |
| socioeconomic status | 0.83 | 0.022 | -7.290 | 0.000 | 0.79 | 0.87 |  |
| family size | 0.99 | 0.021 | -0.270 | 0.788 | 0.95 | 1.04 |  |
| birth order | 1.01 | 0.058 | 0.100 | 0.919 | 0.90 | 1.13 |  |
| birth order² | 1.00 | 0.006 | 0.390 | 0.694 | 0.99 | 1.01 |  |
| intercept | 0.50 | 0.079 | -4.390 | 0.000 | 0.36 | 0.68 |  |

¹ linear regression analyses, ² discrete-time event-history analyses, ³ binary logistic regression analyses, ^4^ multinomial logistic analyses

Coef. = beta coefficient; O.R. = odds ratio; R.R.R = relative risk ratio, S.E. = standard error; C.I. = confidence interval

Age at first birth is measured in months; all other ages are in years
